# *Clostridium Scindens* Protects Against Vancomycin‐Induced Cholestasis and Liver Fibrosis by Activating Intestinal FXR‐FGF15/19 Signaling

**DOI:** 10.1002/advs.202406445

**Published:** 2024-12-16

**Authors:** Jintao Xiao, Yanliang Hou, Xingyang Luo, Yuhao Zhu, Wenhu Li, Bingbing Li, LinXiang Zhou, Xia Chen, Ying Guo, Xiaomei Zhang, Haiyue He, Xiaowei Liu

**Affiliations:** ^1^ Department of Gastroenterology Xiangya Hospital Central South University Changsha Hunan 410008 China; ^2^ Department of Clinical Laboratory Xiangya Hospital Central South University Changsha Hunan 410008 China; ^3^ Department of Clinical Pharmacology Xiangya Hospital Hunan Key Laboratory of Pharmacogenetics Central South University Changsha Hunan 410008 China; ^4^ National Clinical Research Center for Geriatric Disorders Xiangya Hospital Central South University Changsha Hunan 410008 China

**Keywords:** cholestatic liver diseases, *Clostridium scindens*, FXR‐FGF15/19 signaling, gut microbiota, liver fibrosis, vancomycin

## Abstract

Primary sclerosing cholangitis (PSC) is characterized by abnormal bile acid metabolites and altered gut microbiota, with no effective treatments available. Vancomycin, a glycopeptide antibiotic, has emerged as a promising candidate. However, the mechanism by which vancomycin impacts the progression of PSC remains unknown. Mice treated with vancomycin exhibit increased hepatic collagen deposition and injury, due to the inhibition of intestinal FXR‐FGF15/19 axis and the elevation of bile acid levels. These effects are associated with the reduction in *Clostridia XIVa*, especially *Clostridium scindens* (*C. scindens*). Gavage of *C. scindens* alleviates vancomycin‐induced bile acid accumulation and liver fibrosis via activating intestinal FXR‐FGF15/19 signaling. Similar effects are observed in mice treated with engineered Escherichia coli Nissle 1917 that are capable of expressing bile acid 7α‐dehydratas (BaiE) from *C. scindens* (EcN‐BaiE). Activating intestinal FXR‐FGF15/19 signaling by fexaramine (Fex) or recombinant protein FGF19 reverse vancomycin‐induced liver injury and fibrosis. These results demonstrate that long‐term oral vancomycin exacerbates cholestatic liver injury, while *C. scindens* mitigates this effect by activating the intestinal FXR‐FGF15/19 signaling pathway. This underscores the importance of monitoring bile acid levels in PSC patients receiving vancomycin treatment and suggests that *C. scindens* may serve as a potential therapeutic approach for PSC patients.

## Graphical abstract

Vancomycin administration disrupts the balance of primary and secondary bile acids by reducing *Clostridium scindens* abundance. Bile acid imbalance inhibits intestinal FXR‐FGF15/19 signaling and promotes hepatic CYP7A1 expression, leading to enhanced bile acid accumulation and exaggerating liver injury and fibrosis. Furthermore, oral gavage of *Clostridium scindens or EcN‐BaiE* alleviates vancomycin‐induced bile acid accumulation and liver fibrosis via activating intestinal FXR‐FGF15/19 signaling.

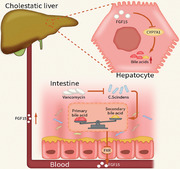

## 1 Introduction

Primary sclerosing cholangitis (PSC) is one of the cholestatic liver diseases (CLD), characterized by elevated liver enzyme parameters and abnormal bile acid metabolites and alterations in the gut microbiota.^[^ ^1^ ^]^ It is well‐established that the gut microbiota‐bile acid axis plays an important role in cholestatic liver injury. Depletion of bacteria using an antibiotics cocktail (ABX) aggravates cholestasis and liver injury in Mdr2^−/−^ mice.^[^ ^2^ ^]^

PSC patients lack effective medical therapy and oral vancomycin, was recently identified as a potential therapy. It has been reported that oral vancomycin can reduce alkaline phosphatase (ALP) activity, and serum bilirubin and improve Mayo PSC risk score in a pilot study.^[^ ^3^ ^]^ However, another study implies that depletion of vancomycin‐sensitive microbiota, such as Lachnospiraceae, exacerbates liver damage by decreasing the production of short‐chain fatty acids (SCFA), while neomycin has minimal effect on liver injury.^[^ ^4^ ^]^ Vancomycin, as a broad‐spectrum antibiotic, needs further evaluation to assess its efficacy and safety in the treatment of PSC. Additional research is required to ascertain whether vancomycin has beneficial or detrimental effects on bile acid metabolism, liver injury, and fibrosis, as well as to identify the specific species of microbiota involved.

A clinical trial reveals that obese patients treated with oral vancomycin exhibit a decreased abundance of *Clostridium XIVα* and increased levels of primary bile acids in plasma, which suggests a potentially adverse effect on cholestasis.^[^ ^5^ ^]^ *Clostridia XIVa* plays a significant role in combating *Clostridioides difficile* infection, regulating the intestinal immune system, and affecting bile acid and lipid metabolism, primarily through its metabolites such as SCFA or the conversion of primary bile acids to secondary bile acids.^[^ ^6^ ^]^ *Clostridium scindens (C. scindens)* is a high‐profile representative of *Clostridium XIVα*, encoding the bile acid‐induced (bai) operon that carries out 7‐alpha‐dehydroxylation.^[^ ^7^ ^]^ However, whether vancomycin regulates the progression of PSC through *Clostridium XIVα* still requires further experimental elucidation.

Herein, we investigated the role of vancomycin in regulating bile acid metabolism and liver fibrosis using PSC mouse models. Our results demonstrated that long‐term vancomycin treatment exacerbates cholestasis and liver fibrosis in both partial bile duct ligation (pBDL)‐induced and 1% cholic acid (CA) diet‐induced PSC mouse models. The reduced abundance of fecal *Clostridium XIVα* induced by oral vancomycin contributed to aggravated bile acid accumulation in the liver by inhibiting intestinal FXR‐FGF15/19 signaling. Remarkably, the gavage of *C. scindens* reversed vancomycin‐induced bile acid accumulation and liver fibrosis by activating intestinal FXR‐FGF15/19 signaling. Our findings emphasized the necessity of monitoring bile acid levels in PSC patients undergoing vancomycin treatment and revealed a protective role of *C. scindens* in the progression of PSC.

## 2 Results

### 2.1 Vancomycin Treatment Aggravates pBDL and 1%CA Diet‐Induced Liver Injury and Fibrosis

**Figure 1:**
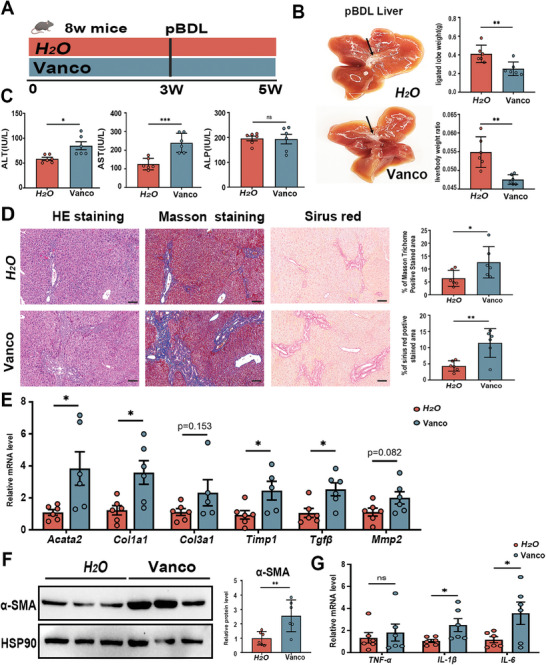
Vancomycin treatment aggravates pBDL‐induced liver injury and fibrosis. A) Experimental scheme: C57BL/6J mice were pretreated with vancomycin or regular water for 3 weeks and followed by pBDL operation for 2 weeks. Vanco‐vancomycin. B) Representative liver images and arrowheads indicate the ligation site. Liver/body weight ratio. C) Serum ALT, AST and ALP levels. D) Representative images of liver specimens stained with hematoxylin and eosin, Masson trichrome, and Sirius red (scale bar, 100 µm). The bar graph represents the average percentage of Masson trichrome or Sirius red positive area per field. E) Hepatic mRNA expression of liver fibrosis‐related genes. F) Representative immunoblots (left panel) and quantification (right panel) of α‐SMA in liver tissue. G) Hepatic mRNA expression of liver inflammation‐related genes. Data were expressed as mean ± SEM, *n* = 6 individuals/group. ^*^p < 0.05, ^**^p < 0.01, ^***^p < 0.001; ns: no significance.

When mice were pretreated with vancomycin for 3 weeks and followed by pBDL operation for 14 days (**Figure** **1**A), we observed a decrease in liver/body weight ratio and increases in serum alanine aminotransferase (ALT) and aspartate transferase (AST), while no difference was noted in serum ALP levels (Figure 1B,C). Increased necrosis of liver cells and collagen deposition in vancomycin‐treated mice were as further evidenced by increases in Sirius red positive area and Masson's trichrome staining positive area (Figure 1D). Consistent with this, we also found evaluated mRNA expression of fibrosis‐associated genes (*Acta2*, *Col1a1*, *Timp1*, and *Tgf‐β*) in vancomycin‐treated mice, as well as an increase in α‐SMA protein levels (Figure 1E,F). Vancomycin‐treated mice also exhibited increased mRNA levels of genes that related to hepatic inflammation, such as *Il‐1β*, and *Il‐6* (Figure 1G). These results established that vancomycin promotes hepatic collagen deposition and hepatic stellate cell (HSC) activation in the pBDL‐induced mouse model.

To further confirm the contribution of vancomycin treatment to cholestasis and fibrosis, we built up another PSC mouse model using a 1% CA diet. Mice were administrated with water containing 0.5 g L^−1^ vancomycin or regular water for 6 weeks, with free access to a regular diet or a diet supplemented with 1% CA (Figure S1A, Supporting Information). In keeping with those findings we observed 1% CA diet‐fed mice with vancomycin treatment exhibited increased ALT, AST, and ALP levels in serum (Figure S1B, Supporting Information). Moreover, body weight and liver weight were decreased in 1% CA diet‐fed mice with vancomycin treatment (Figure S1C, Supporting Information). We also found increased collagen synthesis and deposition in 1% CA diet‐fed mice with vancomycin treatment, as evidenced by increased Sirius red positive area and protein level of α‐SMA (Figure S1D,E, Supporting Information). 1% CA diet‐fed mice with vancomycin treatment exhibited increased hepatic mRNA expression of *Tnf‐α* (Figure S1F, Supporting Information).

### 2.2 Vancomycin Treatment Exaggerates Bile Acid Accumulation and Inhibits Intestinal FXR‐FGF15 Signaling in pBDL and 1% CA Diet‐Induced Mouse Models

**Figure 2:**
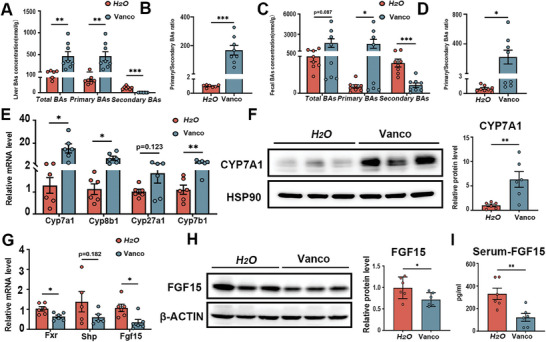
Vancomycin treatment exaggerates bile acid accumulation by inhibiting intestinal FXR‐FGF15 signaling in a pBDL‐induced mouse model. A) Hepatic total, primary and secondary bile acid levels. B) Primary/secondary bile acid ratio in livers. C) Total, primary and secondary bile acid levels in feces. D) Primary/secondary bile acid ratio in feces. E) Hepatic mRNA expression of bile acid synthetic genes. F) Representative immunoblots (left panel) and quantification (right panel) of CYP7A1 in liver tissue. G) Gene expression of *Fxr, Shp* and *Fgf15* in ileum. H) Representative immunoblots (left panel) and quantification (right panel) of FGF15 in ileum tissue. I) Serum FGF15 levels. *n* = 6‐8 individuals/group. Data were expressed as mean ± SEM. ^*^p < 0.05, ^**^p < 0.01, ^***^p < 0.001; ns: no significance.

The characterization of PSC is the abnormal accumulation of bile acids. Therefore, bile acid profiles were detected using HPLC‐MS/MS. Compared to control mice, we observed dramatically increased total and primary bile acids in the liver from vancomycin‐treated mice, along with a decrease in secondary bile acids (**Figure** **2**A). Notably, the primary/secondary bile acid ratio was dramatically increased in the liver of vancomycin‐treated mice compared to control mice (Figure 2B). The Vancomycin treatment markedly increased primary bile acids (CA, TCA, β‐MCA, T‐β‐MCA, CDCA, TCDCA, UDCA, TUDCA) in liver tissue (Figure S2A, Supporting Information). Conversely, most of the secondary bile acids (ω‐MCA, T‐ω‐MCA, HDCA, THDCA, DCA, NorDCA, TDCA, LCA, 7_ketoLCA, ACA) were decreased in the liver from vancomycin‐treated mice (Figure S2B, Supporting Information). Additionally, we also observed increased total bile acid, as well as increased T‐α‐MCA, β‐MCA, T‐β‐MCA, CDCA, TCDCA, and TUDCA in serum from vancomycin‐treated mice, while decreases in ω‐MCA, DCA and TDCA (Figure S2C–E, Supporting Information). Consistent with changes observed in mice treated with vancomycin, the bile acid profiles of serum from CLD patients also showed increased total and primary bile acids, along with increased primary/secondary bile acid ratio, compared to healthy controls (Tables S1–S2, Supporting Information).

Vancomycin is a glycopeptide antibiotic, that primarily targets gram‐positive bacteria. Therefore, we also investigated bile acid concentrations in feces. Consistent with the alterations observed in hepatic bile acids, we found a trend toward increased total bile acids and significantly increased primary bile acids in feces from vancomycin‐treated mice (Figure 2C). The primary/secondary bile acid ratio was increased in the feces of vancomycin‐treated mice compared to control mice (Figure 2D). As anticipated, we observed elevated levels of TCA, α‐MCA, and T‐β‐MCA in feces from vancomycin‐treated mice, while levels of ω‐MCA, HDCA, DCA, NorDCA, LCA, 6_ketoLCA and 7_ketoLCA were decreased (Figure S2F,G, Supporting Information).

Given the increased bile acid accumulation in vancomycin‐treated mice, we examined the most important enzymes for bile acid synthesis. As expected, we observed increased hepatic mRNA levels of *Cyp7a1*, *Cyp8b1*, and *Cyp7b1* in vancomycin‐treated mice (Figure 2E), as well as an increase in the protein level of CYP7A1 (Figure 2F). It is well‐established that hepatic FXR negatively regulates the transcription of Cyp7a1 through a small heterodimer partner (SHP). Additionally, we observed a trend toward decreased hepatic mRNA level of *Fxr* and significantly decreased hepatic mRNA level of *Shp* in vancomycin‐treated mice (Figure S3A, Supporting Information).

The balance of the bile acid pool relies on both synthesis and transport rate, so we assessed the expression level of major bile acid transporters in the liver and ileum. We observed decreases in hepatic mRNA levels of bile acid transporters, including *Oatp1a1*, *Mdr2*, *Mrp3*, and *Ost‐α*, in vancomycin‐treated mice (Figure S3B, Supporting Information). The mRNA expressions of bile acid transporters in the ileum, such as *Asbt*, *Ibabp*, and *Ost‐α*, were also decreased in vancomycin‐treated mice (Figure S3C, Supporting Information). These findings indicate that vancomycin exacerbates bile acid accumulation by enhancing bile acid synthesis rate and inhibiting bile acid transport rates. Antibiotic treatment has been reported to be involved in intestinal and liver diseases by regulating multiple bile acid‐dependent transcriptional factors, including FXR, VDR, PXR, and so on.^[^ ^8^ ^]^ We only observed decreased mRNA levels of *Fxr* and downstream gene *Fgf15* in the ileum from vancomycin‐treated mice (Figure 2G), while no difference was noted in mRNA expression of *Lxr*, *Pxr*, *Rorα*, *Tgr5*, and *Vdr* (Figure S4, Supporting Information). We also observed a decrease in the protein levels of FGF15 in the ileum from vancomycin‐treated mice, compared to control mice (Figure 2H). Consistently, FGF15 levels in serum were decreased in vancomycin‐treated mice (Figure 2I). We also found enhanced bile acid accumulation and inhibition of intestinal FXR‐FGF15/19 signaling by vancomycin administration in the absence of a disease model (sham operation) (Figure S5, Supporting Information). To further verify the effect of vancomycin on the progression of CLD, we conducted a pBDL operation and subsequently treated it with vancomycin or vehicle. Consistent with previous findings, this model demonstrated increased collagen deposition and bile acid accumulation in mice exposed to vancomycin following disease induction (Figure S6, Supporting Information). Although vancomycin treatment resulted in increased fecal and hepatic bile acid concentration, intestinal barrier function was unaffected, as evidenced by no difference in pathological examination and the expression of tight junction proteins (Figure S7, Supporting Information). These findings demonstrate that vancomycin inhibits ileum FXR‐FGF15/19 signaling, thereby promoting bile acid synthesis through increased hepatic CYP7A1 expression.

Consistently, we observed dramatically increased total bile acid levels in the serum of vancomycin‐treated mice fed with a 1% CA diet (Figure S8A, Supporting Information). The livers of 1% CA diet‐fed mice with vancomycin treatment also exhibited decreased mRNA expression of *Fxr* in liver tissue (Figure S8B, Supporting Information). The protein levels of CYP7A1 were increased in the livers and the protein levels of FGF15 were decreased in the ileum from 1% CA diet‐fed mice with vancomycin treatment (Figure S8C,D, Supporting Information). These results further implicate that vancomycin aggravates cholestasis and fibrosis via inhibiting intestinal FXR‐FGF15/19 signaling and increasing hepatic CYP7A1 expression.

### 2.3 Vancomycin Treatment Leads to a Reduction in Microbiota Diversity and Suppresses the Abundance of *Clostridium XIVα*

**Figure 3:**
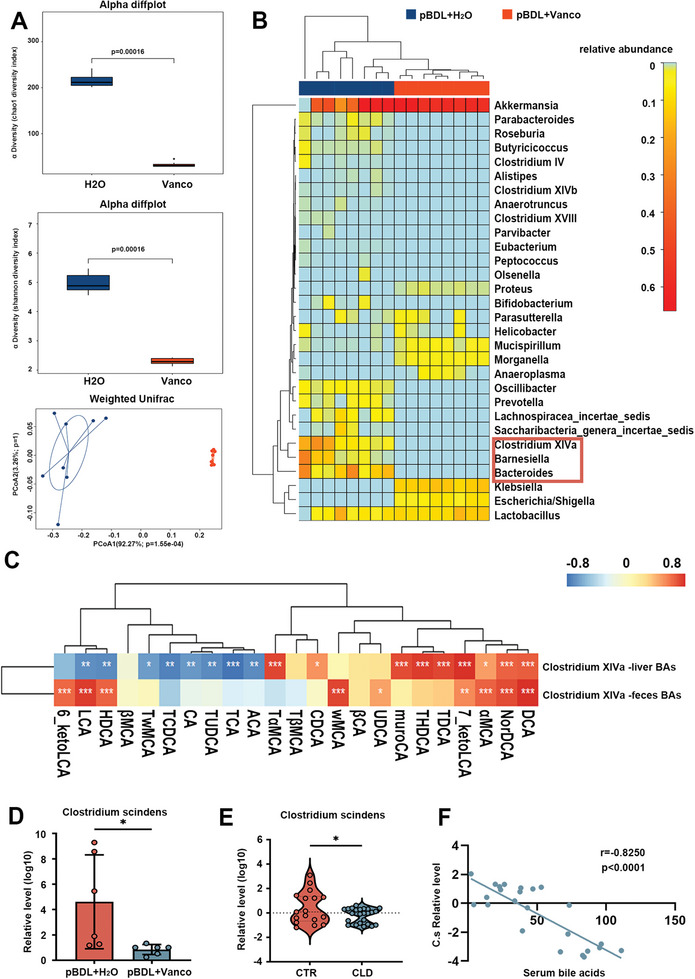
Vancomycin treatment decreases the diversity of microbiota and suppresses the abundance of *Clostridium XIVα*. Fecal samples from each mouse were collected for 16S rRNA sequencing analysis. A) Chao1 and Shannon indexes to analyze intestinal microbial α diversity to reflect species diversity and richness. PCoA evaluation showed microbiome variation in two groups based on Weighted Unifrac. B) The heat maps showed the relative abundance of genus levels in the feces. C) Spearman correlation heatmap analysis was conducted between the *Clostridium XIVα* and the hepatic/fecal BA profile in feces. D) The relative level of *Clostridium scindens* in the feces of mice. *n* = 6 individuals/group. E) The relative level of *Clostridium scindens* in feces of healthy controls (*n* = 17) and CLD patients (*n* = 24). CTR, control; CLD, cholestatic liver disease. F) The correlation between the abundance of *C. scindens* and serum bile acid levels in CLD patients (*n* = 24) Data were expressed as mean ± SEM. ^*^p < 0.05, ^**^p < 0.01, ^***^p < 0.001; ns: no significance.

Gut microbiota is essential to maintain bile acid homeostasis, mainly relying on the conversion of primary bile acids to secondary bile acids. Furthermore, altered gut microbiota has been implicated in the pathogenesis of PSC. Feces from vancomycin‐treated and control mice were analyzed by 16S rDNA. We observed lower alpha diversity in vancomycin‐treated mice than in control mice (**Figure** **3**A). The results of 16S rRNA sequencing revealed decreased abundances of *Clostridium XIVα*, *Barnesiella*, and *Bacteroides* in vancomycin‐treated mice, while *Klebsiella*, *Escherichia/Shigella*, *Mucispirillum*, and *Morganella* were increased (Figure 3B). Our study aims to identify potential probiotics involved in vancomycin‐induced cholestatic liver injury, with *Clostridium XIVα*, *Barnesiella*, and *Bacteroides* considered as candidate species. The gut microbiota participates in bile acid homeostasis by converting primary bile acids into secondary bile acids, with *Clostridium XIVα* recognized as the primary bacterial cluster involved in this metabolic pathway.^[^ ^9^ ^]^ However, both *Barnesiella* and *Bacteroides*, as fiber‐degrading bacteria, primarily play roles in the intestinal barrier, immunity, and brain health.^[^ ^10^ ^]^ Therefore, we next investigated whether changes induced by vancomycin in *Clostridium XIVα* correlate with bile acids, Spearman correlation analysis was conducted. We found that the hepatic concentration of T‐α‐MCA, CDCA, MuroCA, THDCA, TDCA, 7_ketoLCA, α‐MCA, NorDCA and DCA has significantly positive correlations with the abundance of *Clostridium XIVα*, while hepatic concentration of LCA, HDCA, T‐ω‐MCA, TCDCA, CA, TUDCA, TCA and ACA has significantly negative correlations with it. In addition, the fecal concentration of 7_ketoLCA, UDCA, LCA, DCA, NorDCA,6_ketoLCA, HDCA, ω‐MCA, and α‐MCA had significantly positive correlations with the abundance of *Clostridium XIVα* (Figure 3C). This correlation may be attributed to the 7‐alpha‐dehydroxylation enzyme activity in *Clostridium XIVα*. *Clostridium scindens* (*C. scindens*) is a predominant bacterium in *Clostridium XIVα*, known for its high levels of bile acid 7‐dehydroxylating activity.^[^ ^11^ ^]^ qPCR results showed decreased levels of *C. scindens* in vancomycin‐treated mice and CLD patients, compared to controls (Figure 3D,E). We also analyzed the correlation between *C. scindens* and serum bile acid concentrations. The abundance of *C. scindens* was negatively correlated with serum bile acid levels in CLD patients (Figure 3F). These findings suggest that vancomycin treatment alters gut microbiota, especially decreasing the abundance of *C. scindens*, which may contribute to bile acid accumulation and liver fibrosis.

### 2.4 *C. scindens* Alleviates Vancomycin‐Induced Bile Acid Accumulation and Liver Fibrosis via Activating Intestinal FXR‐FGF15/19 Signaling

**Figure 4:**
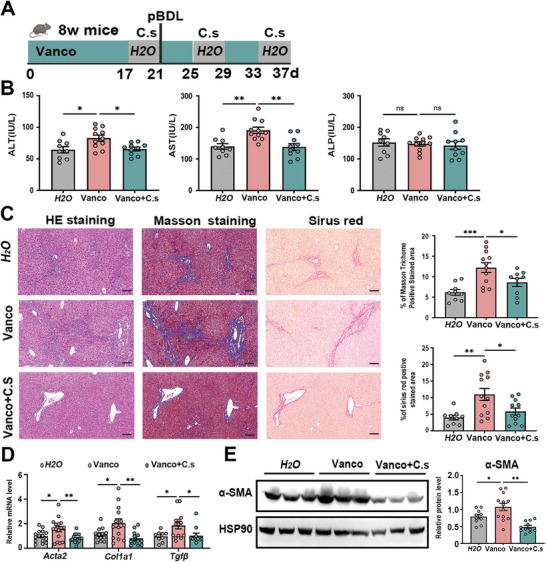
*C. scindens* alleviates vancomycin‐induced liver fibrosis. C57BL/6J mice were pretreated with vancomycin or regular water, followed by colonization with *C. scindens* or vehicle, and subjected to pBDL operation as illustrated in A). B) Serum ALT, AST and ALP levels. C) Representative images of liver specimens stained with hematoxylin and eosin, Masson's trichrome, and Sirius red (scale bar, 100µm). The bar graph represents the average percentage of Masson trichrome or Sirius red positive area per field. D) Hepatic mRNA expression of liver fibrosis‐related genes. E) Representative immunoblots (left panel) and quantification (right panel) of α‐SMA in liver tissue. *n* = 10–13 individuals/group. Data were expressed as mean ± SEM. ^*^p < 0.05, ^**^p < 0.01, ^***^p < 0.001.

To confirm whether vancomycin aggravates bile acid accumulation and liver fibrosis by decreasing the abundance of *C. scindens*. C57BL/6J mice were exposed to water containing 0.5 g L^−1^ vancomycin for 3 weeks, followed by daily gavage of *C. scindens* or vehicle, and then administrated to pBDL operation (**Figure** **4**A). Consistent with previous results, vancomycin treatment led to elevated serum ALT and AST levels, and *C. scindens* gavage reduced the vancomycin‐induced ALT and AST levels in serum (Figure 4B). Histologic analysis revealed decreased collagen synthesis and deposition in the *C. scindens* group, with decreases in Sirius red positive area, and Masson's trichrome staining positive area (Figure 4C). Consistently, we also found decreased mRNA levels of *Acta2*, *Col1a1* and *Tgf‐β* after gavage of *C. scindens*, along with decreased α‐SMA protein level (Figure 4D,E). Taken together, these results indicate that restoration of *C. scindens* alleviates vancomycin‐induced hepatic fibrosis.

**Figure 5:**
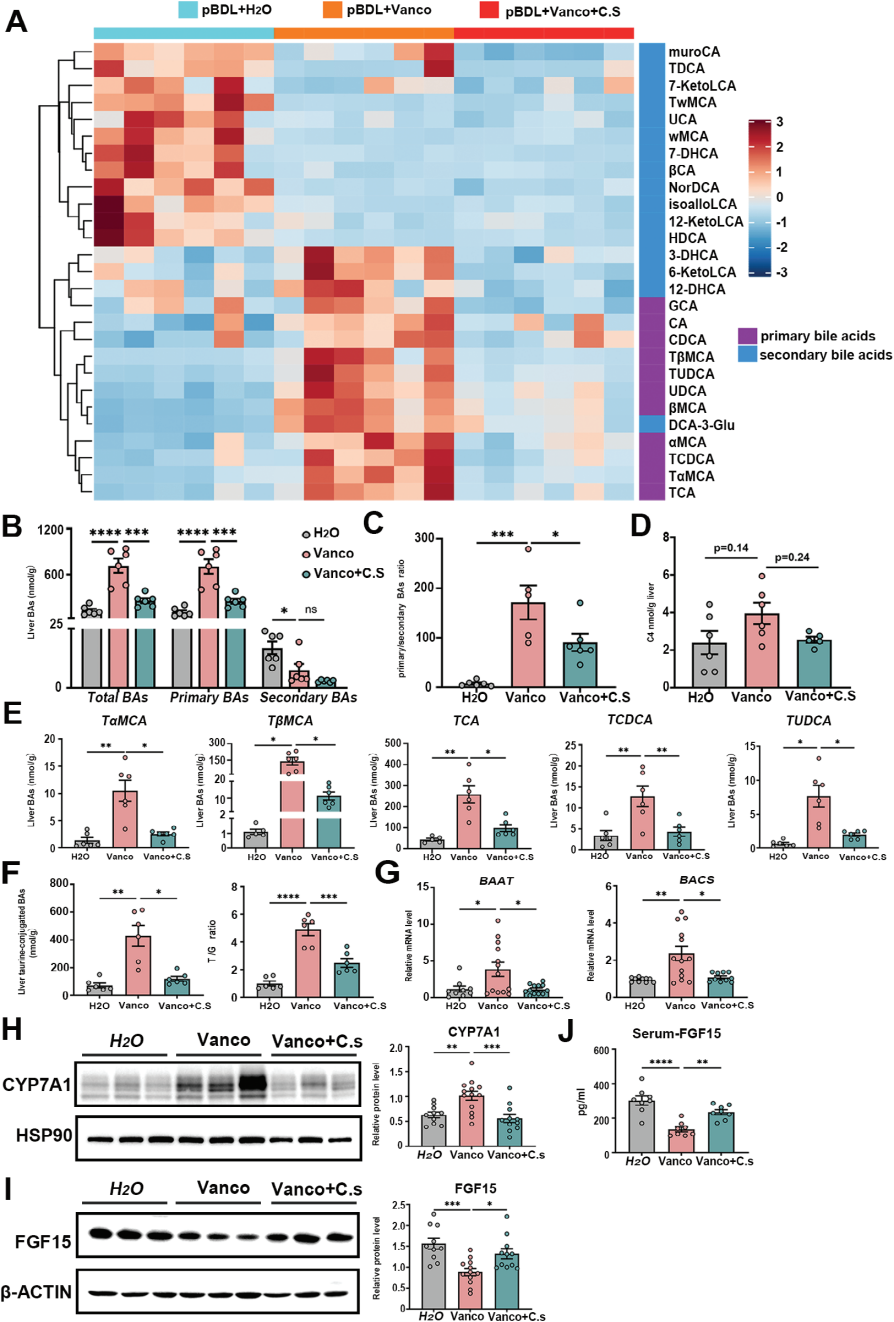
*C. scindens* alleviates vancomycin‐induced bile acid accumulation via activating intestinal FXR‐FGF15/19 signaling. A) Heat map showing changes in bile acid profiles in feces among three groups. B) Hepatic total, primary, and secondary bile acid levels. C) Primary/secondary bile acid ratio in the livers. D) Concentration of serum 7α‐hydroxy‐4‐cholesten‐3‐one (C4). E) Concentrations of TαMCA, TβMCA, TCA TCDCA, and TUDCA in the liver. F) Concentrations of taurine conjugated‐bile acids and the ratio of taurine to glycine‐conjugated bile acid in the liver. *n* = 6 individuals/group. G) Hepatic mRNA expression of *Baat* and *Bacs*. H) Representative immunoblots (left panel) and quantification (right panel) of CYP7A1 in liver tissue. I) Representative immunoblots (left panel) and quantification (right panel) of FGF15 in ileum tissue. J) Serum FFG15 levels. *n* = 10‐13 individuals/group. Data were expressed as mean ± SEM. ^*^p < 0.05, ^**^p < 0.01, ^***^p < 0.001.

As a 7α‐dehydroxylation bacteria, *C. scindens* could convert primary bile acids to secondary bile acids, we next determined bile acid profiles in the liver and feces from *C. scindens* group mice and control mice. Compared with mice in the vancomycin group, we observed decreased total and primary in the liver from mice in the *C. scindens* group, with a decrease in the primary/secondary bile acid ratio (**Figure** **5**A–C). 7α‐hydroxy‐4‐cholesten‐3‐one (C4) is used as a biomarker for the rate of bile acid synthesis.^[^ ^12^ ^]^ As expected, we observed a trend to increase in hepatic C4 levels in the vancomycin group, and gavage of *C. scindens* had a trend to reduce the C4 level (Figure 5D). Concentrations of five tauro‐conjugated bile acids were decreased in the liver of mice treated with *C. scindens*, including T‐α‐MCA, T‐β‐MCA TCA, TCDCA, and TUDCA (Figure 5E). We observed elevated levels of total taurine‐conjugated bile acids, accompanied by an increased ratio of taurine to glycine‐conjugated bile acids (Figure 5F). Consistently, hepatic mRNA levels of bile acid CoA: amino acid N‐acyltransferase (BAAT) and bile acid‐CoA: amino acid N‐acyltransferase (BACS), in the livers of mice treated with vancomycin compared to control mice. However, these increases were reversed in mice treated with *C. scindens* (Figure 5G). Our in vitro experiments showed that Caco‐2 cells treated with varying concentrations of TCA, TCDCA, and T‐β‐MCA for 48 h exhibited decreased mRNA levels of *Fxr*, *Shp*, and *Fgf15* in a dose‐dependent manner (Figure S9A—C, Supporting Information). Additionally, LX2 cells treated with TCA showed increased mRNA expression of *Acta2*, *Col1a1*, and *Tgf‐β* (Figure S9D, Supporting Information). These distinct bile acids, identified in mice treated with C. *scindens*, inhibited intestinal FXR‐FGF15/19 signaling and promoted HSC activation in vitro. Considering that *C. scindens* limited bile acids accumulation, we next determined the expression of enzymes for bile acids synthesis. Gavage of *C. scindens* reduced the protein level of CYP7A1 compared to the vancomycin group (Figure 5H). As we precious showed, vancomycin treatment inhibited intestinal FXR‐FGF15/19 signaling. We next tested whether the gavage of *C. scindens* could affect the intestinal FXR‐FGF15 axis. Our results showed that the protein level of FGF15 was increased in mice with *C. scindens* gavage, compared to mice in the vancomycin group (Figure 5I). We also observed that vancomycin treatment resulted in decreased serum FGF15 levels, whereas *C. scindens* gavage increased serum FGF15 levels (Figure 5J). These results implicate that restoration of *C. scindens* alleviates vancomycin‐induced bile acid synthesis and accumulation by activating intestinal FXR‐FGF15/19 signaling and inhibiting the expression of hepatic CYP7A1.

### 2.5 The Engineered EcN‐BaiE Alleviates Vancomycin‐Induced Liver Fibrosis via Inhibiting Hepatic CYP7A1 Expression

**Figure 6:**
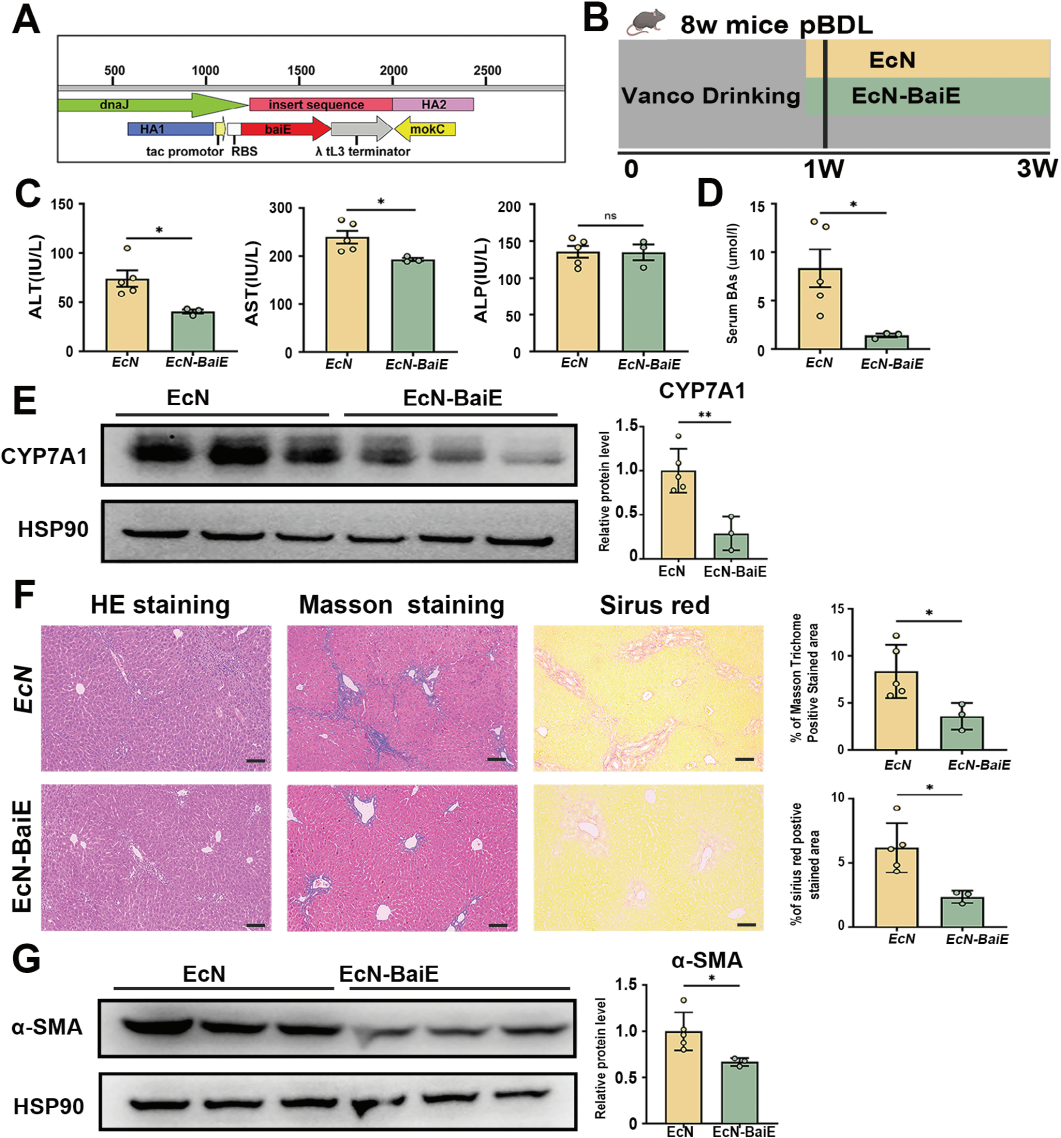
Engineered EcN‐BaiE strain alleviates vancomycin‐induced liver fibrosis and alleviates vancomycin‐induced bile acid accumulation. A) Schematic illustration of genome engineering in the EcN strains. C57BL/6J mice were pretreated with vancomycin or regular water and followed by colonized with EcN‐BaiE or control EcN strains as illustrated in B). C) Serum ALT, AST and ALP levels. D) Serum total bile acid (BA) levels. E) Representative immunoblots (left panel) and quantification (right panel) of CYP7A1 in liver tissue. F) Representative images of liver specimens stained with hematoxylin and eosin, Masson's trichrome, and Sirius red (scale bar, 100µm). The bar graph represents the average percentage of Masson trichrome or Sirius red positive area per field. G) Representative immunoblots (left panel) and quantification (right panel) of α‐SMA in liver tissue. *n* = 3‐5 individuals/group. Data were expressed as mean ± SEM. ^*^p < 0.05, ^**^p < 0.01, ^***^p < 0.001; ns‐no significance.

It is well‐established that the 7‐α‐dehydroxylation strain shares the BaiCD, BaiE, BaiF, BaiG, BaiH, and BaiI genes, which encode enzymes responsible for converting primary bile acids to secondary bile acids.^[^ ^13^ ^]^ Site‐directed mutagenesis of BaiE from *C. scindens* confirmed that baiE is the rate‐determining enzyme in this pathway.^[^ ^14^ ^]^ We constructed engineered bacteria EcN‐BaiE and investigated its function in PSC model mice treated with vancomycin (**Figure** **6**A,B). Mice administrated with EcN‐BaiE stains exhibited decreased serum ALT, AST, and total bile acid (TBA) levels, compared to the EcN group (Figure 6C,D). Similar observations were made with *C. scindens* transplantation, gavage of EcN‐BaiE stains also resulted in reduced protein expression of CYP7A1 in the liver (Figure 6E). Histologic analysis revealed reduced collagen synthesis and deposition in the EcN‐BaiE group, as evidenced by decreases in Sirius red positive area and Masson's trichrome staining positive area, compared to the EcN group (Figure 6F). Consistently, we also found decreased protein levels of α‐SMA after administration of EcN‐BaiE stains (Figure 6G). All these findings demonstrate that bile acid 7α‐dehydratase, which is expressed in *C. scindens*, leads to the inhibition of hepatic CYP7A1 expression, thereby attenuation of liver fibrosis.

### 2.6 Activation of the Intestinal FXR‐FGF15/19 Axis Reverses Vancomycin‐Induced Liver Fibrosis

**Figure 7:**
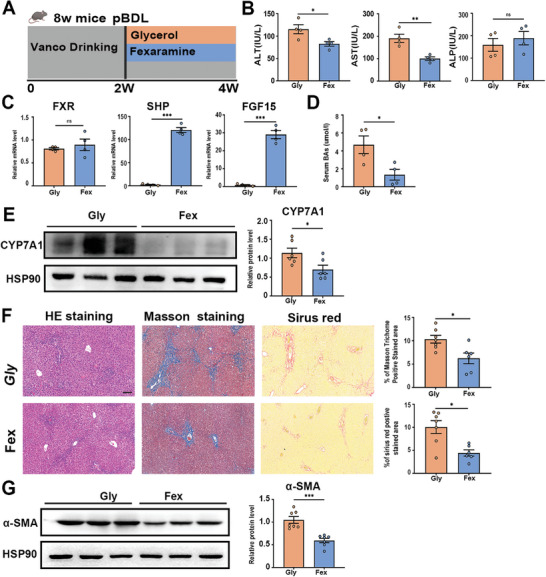
Activation of intestinal FXR‐FGF15/19 axis reverses vancomycin‐induced liver fibrosis. A) Experimental scheme: C57BL/6J mice were pretreated with vancomycin for 2 weeks and followed by pBDL operation, Fexaramine and glycerol were oral gavaged respectively for 2 weeks. B) Serum ALT, AST and ALP levels. C) Gene expression of *Fxr, Shp*, and *Fgf15* in the ileum. D) Serum total bile acid levels. E) Representative immunoblots (left panel) and quantification (right panel) of CYP7A1 in liver tissue. F) Representative images of liver specimens stained with hematoxylin and eosin, Masson's trichrome, and Sirius red (scale bar, 100µm). The bar graph represents the average percentage of Masson trichrome or Sirius red positive area per field. G) Representative immunoblots (left panel) and quantification (right panel) of α‐SMA in liver tissue. *n* = 5‐8 individuals/group. Data were expressed as mean ± SEM. ^*^p < 0.05, ^**^p < 0.01, ^***^p < 0.001; ns‐no significance.

To further confirm the contributions of intestinal FXR‐FGF15/19 to vancomycin‐induced liver fibrosis, we utilized Fexaramine (Fex), an intestinal‐restricted FXR agonist, to activate intestinal FXR in vivo (**Figure** **7**A). We observed decreased ALT and AST levels in serum from Fex‐treated mice (Figure 7B). The mRNA level of downstream genes for FXR activation, such as *Shp* and *Fgf15*, were dramatically increased in the ileum from Fex‐treated mice, compared to control mice treated with vehicle (Figure 7C). We observed decreased TBA level in serum from Fex‐treated mice (Figure 7D). Intestinal FXR‐FGF15/19 signaling represses CYP7A1 in the liver.^[^ ^15^ ^]^ Mice treated with Fex exhibited decreased protein levels of CYP7A1 in the liver (Figure 7E), which may be correlated with the decreased Sirius red positive area, and Masson's trichrome staining positive area (Figure 7F). The protective function of Fex against vancomycin‐induced liver fibrosis was further evidenced by decreased protein levels of α‐SMA (Figure 7G). We also found that the administration of recombinant protein FGF19 also reversed vancomycin‐induced cholestasis and fibrosis by suppressing the expression of CYP7A1 (Figure S10, Supporting Information). These results further implicate that vancomycin‐aggravated liver fibrosis depends on the suppression of intestinal FXR‐FGF15/19 signaling.

## 3 Discussion

Antibiotics, serving as microbiota modulators, are emerging as a promising therapeutic strategy for PSC patients.^[^ ^16^ ^]^ Among them, vancomycin stands out as a promising candidate.^[^ ^17^ ^]^ Although several clinical trials have demonstrated the benefits of vancomycin on ALP and the Mayo risk score, the impact of long‐term vancomycin on cholestasis and fibrosis remains controversial. Inhibition of *Clostridium XIVα* by vancomycin led to decreased bile acid concentrations in the liver of conventional mice, and similar findings were observed in mice with an inulin‐containing diet‐induced HCC model.^[^ ^18^ ^]^ However, increased bile acid levels and enhanced collagen deposition were found in obese patients or HFD‐fed mice with oral vancomycin treatment.^[^ ^5^, ^19^ ^]^ Our results showed the adverse consequence of aggravated cholestasis and liver injury induced by long‐term oral vancomycin due to gut microbiota disturbance, especially the decreased *Clostridium XIVα* abundance, in the mouse models mimicking PSC. This disruption is characterized by a decrease in the ratio of secondary/primary bile acid. The controversial findings regarding the effects of vancomycin on cholestasis and fibrosis may be attributed to different pathological conditions. Our results underscore the importance of monitoring serum bile acid profiles and evaluating hepatic fibrosis levels in PSC patients undergoing oral vancomycin treatment, particularly in those with existing cholestasis.

Gut microbiota is essential to maintain bile acid homeostasis and microbiota‐targeted therapies represent promising strategies for chronic liver disease. Kai  et al found depletion of gut microbiota by ABX promotes bile acid accumulation and liver injury by inhibiting FXR signaling.^[^ ^2^ ^]^ However, the particular bacterial species implicated in the development of liver injury remains unclear. Our results have identified *Clostridium XIVα*, particularly *C. scindens*, as the bacterial species that regulates bile acid metabolism, thereby activating the intestinal FXR‐FGF 15/19 pathway and alleviating vancomycin‐induced cholestatic liver injury. Studies have reported a significant reduction in the abundance of *C. scindens* in nonalcoholic fatty liver disease (NAFLD) and administration of *C. scindens* suppresses the development of NAFLD.^[^ ^20^ ^]^ In our study, we observed similar outcomes where gavage of *C. scindens* restored bile acid homeostasis, as evidenced by reductions in total and primary bile acids in both the liver. The decreases in primary bile acid levels contributed to the alleviation of collagen deposition in the liver. All these findings indicated that targeting 7α‐dehydroxylation clostridial species contributes to the bile aid homeostasis and could be a therapeutic approach for PSC patients. However, C*. scindens* has been implicated in facilitating the onset and progression of irritable bowel syndrome.^[^ ^18a^ ^]^ Therefore, the safety and efficacy of clinical use of single‐strain probiotics like *C. scindens* in clinical settings need to be further evaluated through large randomized controlled trials.

The engineered bacteria, belonging to live biotherapeutic products, have been approved to be effective in the treatment of multiple diseases. EcN is widely used as a safety vehicle for engineered biotherapeutics due to its ability to adapt to the environment of the human digestive tract.^[^ ^21^ ^]^ We constructed the engineered EcN that overexpresses 7α‐dehydratase (baiE) of *C. scindens*.^[^ ^14^ ^]^ Gavage of EcN‐BaiE attenuated cholestatic liver injury and fibrosis by reducing the expression of CYP7A1 in the liver. With recent advancements in molecular biology, the engineered EcN with the overexpression of baiE may be a candidate for the treatment of PSC patients.

The gut microbiome influences bile acid profiles, which further impact liver disease progression by affecting intestinal homeostasis.^[^ ^22^ ^]^ No significant differences were observed in the intestinal barrier function of mice treated with vancomycin in our study. We only observed inhibition of intestinal FXR‐FGF15/19 signaling with oral vancomycin in mouse models, while the other bile acid‐related signaling pathways (such as VDR, PXR, TGR5, and RORα) remained unchanged. Intestinal FXR‐FGF15/19 signaling plays a protective role in chronic liver disease, including NAFLD/NASH, CLD, and liver cancer.^[^ ^23^ ^]^ This signaling pathway plays a crucial role in maintaining bile acid and gut microbiota homeostasis through negative feedback regulation of bile acid synthesis. The effects of vancomycin on the FXR‐FGF15 pathway and bile acid metabolism remain controversial. After administering vancomycin, conventional mice exhibit activation of the intestinal FXR‐FGF15/19 signaling pathway and a decrease in the levels of taurine‐conjugated bile acids.^[^ ^18a^ ^]^ However, another study shows inhibition of intestinal FXR‐FGF15/19 signaling and evaluation of hepatic bile acid concentrations in C57BL/6J mice treated with vancomycin.^[^ ^24^ ^]^ Our findings observed inhibition of intestinal FXR‐FGF15/19 signaling with oral vancomycin, thereby promoting the hepatic CYP7A1 expression and enhancing hepatic taurine‐conjugated bile acids accumulation. Moreover, intestinal‐specific agonist Fex and recombinant FGF19 protein could attenuate bile acid accumulation and reverse vancomycin‐induced liver fibrosis. In addition, gavage of *C. scindens* activated intestinal FXR‐FGF15/19 signaling in mouse models. These rescue experiments provide compelling evidence to support the findings that vancomycin aggravates cholestasis and liver fibrosis by inhibiting intestinal FXR‐FGF15/19 signaling.

PSC is an autoimmune disease characterized by the narrowing of medium/large bile ducts in both intra and extrahepatic biliary trees, resulting in cholestasis and fibrosis.^[^ ^25^ ^]^ However, there is currently no ideal mouse model that fully mimics the pathogenicity of human PSC. The BDL mouse model is a commonly used experimental model of cholestasis and closely mirrors the histopathological features observed in human cholestasis.^[^ ^26^ ^]^ It is used to elucidate the pathways involved in the progression of PSC.^[^ ^27^ ^]^ Unlike the BDL mouse model, the pBDL mouse model involves ligating only the left hepatic bile duct, which makes it more relevant to human bile duct obstruction.^[^ ^28^ ^]^ Most CLDs, including PSC, have elevated bile acid levels with subtle differences, in which PSC patients demonstrated dramatically increased levels of CA and CDCA.^[^ ^29^ ^]^ CDCA is recognized as the most efficient FXR agonist, which can alleviate cholestasis and liver injury.^[^ ^30^ ^]^ CA is a weak FXR agonist, and a diet supplemented with 1% CA (w/w) induces cholestasis and fibrosis in mice.^[^ ^31^ ^]^ Therefore, we used pBDL and 1% CA diet mouse models to investigate the effect of oral vancomycin on cholestasis and liver fibrosis.

This study has some limitations. First, Several single‐center, small‐sample clinical studies have shown that vancomycin improves serum ALP levels in PSC patients. We observed no difference in serum ALP levels after vancomycin treatment in mouse models. A multicenter, large‐sample clinical study should be conducted to confirm the effects of vancomycin on serum ALP levels. Second, Except for bile acids, *C.scindens* has been reported to regulate SCFA, steroid hormones, and other metabolites. Further studies are needed to determine whether *C.scindens* alleviates cholestatic liver injury and fibrosis by regulating other metabolites and signaling pathways. Thirdly, these mouse models in our study do not fully replicate the complex pathogenesis of human PSC. Clinical trials are necessary to ascertain whether oral vancomycin exaggerates cholestasis and liver fibrosis in PSC patients.

Taken together, our findings demonstrated that oral vancomycin aggravates cholestasis and liver fibrosis by reducing the abundance of 7α‐dehydroxylation clostridial species, especially *C. scindens*. *C. scindens* plays an essential role in bile acid homeostasis by activating intestinal FXR‐FGF15/19 signaling, which leads to the inhibition of hepatic CYP7A1 expression. These findings underscore the importance of monitoring bile acid levels and the abundance of 7α‐dehydroxylation clostridial species in PSC patients treated with vancomycin or other antibiotics. Our study also indicated that *C. scindens* or engineered EcN‐BaiE could be potential therapeutic strategies for PSC patients.

## 4 Experimental Section

### Animal Studies

C57BL/6J mice were housed in a barrier facility on a 12 h light/dark cycle(22 ± 2 °C). Seven‐week‐old mice were divided randomly into 5 mice/cages and housed for 1 week to adjust to the environment. They were then assigned to the H_2_O or vancomycin‐treatment group. Mice in the vancomycin group were provided with drinking water containing vancomycin (0.5 g L^−1^, Sigma‐Aldrich, St. Louis, MO, USA) for 3 weeks, with fresh antibiotic water replaced every other day. After 3 weeks, mice were administrated to either a sham or pBDL operation and received continuous vancomycin treatment for another 2 weeks. In rescue experiments, mice were exposed to water containing vancomycin (0.5 g L^−1^) for 3 weeks, followed by daily gavage of *C. scindens* (10^10^ CFU per mouse) (American Type Culture Collection (ATCC), Manassas, VA, USA) (10^10^ CFU per mouse) or vehicle for 4 days. Subsequently, all mice were administrated to pBDL, repeated with 4 days of vancomycin treatment and 4 days of *C. scindens*/EcN‐BaiE or vehicle. The mice were euthanized 16 days after pBDL and liver tissues were fixed in formalin for histology.

Following 2 weeks of vancomycin treatment, all mice were subjected to pBDL and then half the mice were gavaged with an intestine‐restricted FXR agonist fexaramine (FEX)/ human fibroblast growth factor‐19 recombinant protein (FGF19) /EcN‐BaiE or vehicle (MedChemExpress, Shanghai, China). In selected experiments, after 2 weeks of oral vancomycin treatment, mice then had free access to a diet supplemented with 1% CA (Feed‐Science, Nanjing, China) and continuously received vancomycin for 4 weeks. Animal use and euthanasia protocols were approved by the Institutional Animal Care and Use Committee of the Xiangya Hospital of Central South University (Ethical Approval No. CSU‐2022‐0061).

### Microbial BaiE‐Expressing E.coli Strain Construction and Oral Administration In Vivo

*Escherichia coli* Nissle 1917 (EcN) was purchased from ATCC (Manassas, VA, USA). The information and coding sequences of the BaiE (bile acid 7alpha‐dehydratase) gene of *C.scindens* were extracted from the NCBI database and optimized these genes for expression in EcN. The region encoding of the BaiE enzyme was cloned into sgRNA/Cas9 cloning plasmid and then introduced into EcN through heat stimulation to create the engineered bacteria (EcN‐BaiE). Colonies were selected on LB agar containing kanamycin (50 µg mL^−1^). Finally, pCP20 was utilized to eliminate the kanamycin resistance cassettes, resulting in the generation of the final antibiotic‐free wild‐type and EcN‐BaiE strains.

Wild‐type and EcN‐BaiE strains were cultured with LB and incubated on a shaker at 37 °C overnight. The Wild‐type and EcN‐BaiE strains were then harvested by centrifugation at 3000–5000 × g, washed three times with sterile PBS, and resuspended in sterile ice‐cold PBS (the final concentration of ≈10^11^ CFU mL^−1^).

### Human Samples

Fecal and serum samples were obtained from 17 healthy controls and 24 CLD patients, who were recruited from the Xiangya Hospital of Central South University between 2020 and 2023. The CLD patients and healthy controls were matched based on age and sex. All the participants confirmed that they had not taken any antibiotics within the preceding 3 months before sample collection. This study was approved by the ethics committee of Xiangya Hospital of Central South University (Ethical Approval No. 202008140).

### Analysis of Serum Alanine Aminotransferase (ALT), Aspartate Transaminase (AST), and Alkaline Phosphatase (ALP)

Serum ALT, AST, and ALP levels were assessed using a colorimetric enzymatic assay kit according to the manufacturer's instructions (Sigma‐Aldrich, St. Louis, MO, USA). In brief, 100 µL of the Master Reaction Mix was prepared for each well. Subsequently, 100 µL of the Master Reaction Mix was added to each of the standard, positive control, and test wells (5 µL well^−1^). The wells were mixed and then incubated at room temperature for 3 min. The initial measurement was taken at 570 nm wavelength, followed by further incubation at 37 °C with measurements taken every 5 min. ALT/AST/ALP activity was analyzed according to the manufacturer's instructions.

### Measurement of Serum Total Bile Acid (TBA) Concentrations

Serum TBA concentrations were detected using a fluorometric detection kit according to the manufacturer's instructions (Sigma‐Aldrich, St. Louis, MO, USA). In brief, prepare 250 µL of 80 µm sodium cholate (20 µL Standard + 230 µL ultrapure water) for the Internal Standard. Transfer 20 µL of serum to each of the three wells (Sample, Internal Standard, Sample Blank). Add 5 µL ultrapure water to the Sample and Sample Blank wells, and 5 µL Internal Standard to the Internal Standard well. For the Internal Standard and Sample wells, prepare the Working Reagent by mixing 75 µL Assay Buffer, 8 µL NAD, 4 µL Probe, 1 µL Enzyme A, and 1 µL Enzyme B. For the Sample Blank wells, prepare the Blank Reagent by mixing 75 µL Assay Buffer, 8 µL NAD, 4 µL Probe, and 1 µL Enzyme B (no Enzyme A). Add 80 µL of Working Reagent to the Internal Standard and Sample wells, and 80 µL of Blank Reagent to the Sample Blank wells. Mix by tapping, incubate for 20 min in the dark and read fluorescence intensity at 530 nm excitation and 585 nm emission.

### Measurement of Serum FGF15 Levels

Serum FGF15 levels were detected using an Elisa kit according to the manufacturer's instructions (Cloud‐Clone Crop, Wuhan, China). Add 100 µL of each dilution of standard, blank, and serum into the appropriate wells. Incubate covered with a plate sealer for 1 h at 37 °C. Remove the liquid from each well without washing. Add 100 µL of Detection Reagent A working solution to each well, cover with the plate sealer, and incubate for 1 h at 37 °C. Aspirate the solution and wash each well with 350 µL of 1× Wash Solution, repeating 3 times. Add 100 µL of Detection Reagent B working solution to each well, cover, and incubate for 30 min at 37 °C. Repeat the aspiration/wash process 5 times. Add 90 µL of Substrate Solution to each well and incubate for 15 min at 37 °C, while protecting from light. Add 50µL of Stop Solution to each well, causing the liquid to turn yellow. Ensure no water drops or fingerprints on the plate bottom and no bubbles on the liquid surface. Immediately conduct measurement at 450nm using a microplate reader.

### Histopathology

The liver and ileum tissues were fixed in 4% neutralized formalin for 2 d, followed by processing and paraffin embedding. Sections were then subjected to deparaffinization and stained with Hematoxylin and Eosin (H&E), picro‐sirius or biebrich scarlet‐acid fuchsin solution. Slides were visualized using a Zeiss Axio Imager M1 microscope (Carl Zeiss AG, Oberkochen, Germany).

### Immunoblot Analysis

Tissue (20–40 mg) was homogenized in RIPA buffer containing Protease Inhibitor Cocktail and Phosphate Inhibitor Cocktail Tablets (Roche, Indianapolis, IN, USA), using a homogenizer. Protein concentrations were measured using the BCA Protein Assay Kit (Thermo Fisher Scientific, Waltham, MA, USA). 20–30 µg of protein was separated by SDS‐PAGE gel and then transferred onto a polyvinylidene difluoride membrane. The membranes were blocked for 1 h using 5% BSA w/v dissolved in TBST v/v, and then incubated with primary antibodies overnight at 4 °C. Blots were then probed with secondary antibodies (Sigma‐Aldrich, St. Louis, MO, USA) for 1 h, and detected the signal according to the manufacturer's recommendations (Thermo Fisher Scientific, Waltham, MA, USA). All antibodies are listed in the Table S3 (Supporting Information).

### Quantitative PCR Analysis

According to the manufacturer's instructions, total RNA was extracted from liver tissue or cells using Trizol (Thermo Fisher Scientific, Waltham, MA, USA). 1 µg of RNA was treated with DNase I (Invitrogen, Waltham, MA, USA) and incubated at 37 °C for 30 min, then used as a template for cDNA synthesis using a High‐Capacity cDNA Reverse transcription Kit (Applied Biosystems, Foster City, CA, USA). Power SYBR Green PCR Master Mix (Fisher Scientific, Springfield Township, NJ) was used to quantify relative mRNA expression levels (Fisher Scientific, Springfield Township, NJ) on a QuantStudio 7 Flex Real‐Time PCR System (Applied Biosystems, Foster City, CA, USA). The expression levels were calculated using the ΔΔCt method according to MIQE guidelines.^[^ ^31^ ^]^ β‐actin or 16S was used for normalization. Primer sequences are listed in Table S4 (Supporting Information).

### Bile Acid Profile Measurement by HPLC‐MS/MS

Liver samples (10 mg) were homogenized in 20 µL ultrapure water and 200 µL acetonitrile/methanol (v/v = 8:2) solution containing 10 µL internal standard using 25 mg precooled grinding beads. Centrifuge at 13 500 rpm for 20 min at 4 °C and lyophilize the supernatant on a freeze dryer. Redissolve samples in 100 µL 1:1 mixture solution of acetonitrile/methanol (v/v = 8:2) and ultrapure water. Centrifuge at 13 500 rpm for 20 min at 4 °C and supernatant was transferred into a new 96‐well plate. Frozen fecal samples were homogenized in 20 µL ultrapure water and 200 µL acetonitrile/methanol (v/v = 8:2) solution containing 10 µL internal standard using 25 mg precooled grinding beads. Centrifuge at 13 500 rpm for 20 min at 4 °C. Collect 20 µL of supernatant and add 80 1:1 mixture solution of acetonitrile /methanol (v/v = 8:2) and ultrapure water. Inject 5 µL of standards or prepared liver or fecal sample or serum to ultra‐performance liquid chromatography coupled to tandem mass spectrometry (UPLCMS/MS) system (ACQUITY UPLC‐Xevo TQ‐S, Waters Corp., Milford, MA, USA). The bile acid profile (Table S5 Supporting Information) of the liver and feces was measured and analyzed using the QuanMET v1.0 software of Metabo‐Profile Co. (Shanghai, China).^[^ ^32^ ^]^

### DNA Extraction from Feces

Total DNA was extracted from feces using EasyPure Stool Genomic DNA Kit according to the manufacturer's instructions (TransGen Biotech Co, Bejing, China). Briefly, 200 mg of feces were homogenized with glass beads in a 1.5 mL tube. Add 1 mL of LB21 and mix well by vortex. Homogenate was then incubated at 70 °C for 10 min and centrifuged at 15 000 g for 2 min. The supernatant was transferred to a new tube and 250 µL of PB21 was added. Mixed them by vortex and incubated on ice for 5 min. Centrifuge at 15 000 g for 2 min and transfer the supernatant to a new tube. Add the same volume of ethanol and BB21 to the supernatant and mix well by vortex. Transfer the mixtures to a spin column and centrifuge at 15 000 g for 30 s. Add 500 µL of CB21 and WB21 to the column in turn and centrifuge at 15 000 g for 30 s. Discard the flow‐through and centrifuge at 15 000 g for 2 min. Place the spin column into a new collect tube and add 100 µL elution buffer to the center of the column. Incubate at room temperature for 1 min and centrifuge at 15 000 g for 2 min to elute the DN.

### 16S rDNA Sequencing

DNA was extracted from 1–2 g feces using a QIAamp DNA Microbiome Kit (QIAGEN, Hilden, Germany) according to the manufacturer's instructions. The extracted DNA samples were sent to Novogene Bio‐Technology Co., Ltd. (Nanjing, China) for sequencing and analysis. All the primers were designed based on the V4 hypervariable regions and 16S rRNA genes were amplified with PCR reaction mix. Then sequencing libraries were prepared using the 16S Library Preparation Protocol from the Illumina Miseq platform. Run the sequencing machine of the Miseq system and obtain FASTQ files. Process the overlapping paired‐end FASTQ files in a data curation pipeline implemented in QIIME 2 version 2017.7.0.

### Cell Line and Reagents

Human hepatic stellate cell line (LX2) and Caco‐2 (an immortalized cell line of human colorectal adenocarcinoma cells) were purchased from ATCC (Manassas, VA, USA). LX2 was cultured in Roswell Park Memorial Institute (RPMI) 1640 medium (Thermo Fisher Scientific, Waltham, MA, USA) supplemented with 10% fetal bovine serum (FBS) and 1% penicillin‐streptomycin (Invitrogen, Waltham, MA, USA). Caco‐2 was cultured in high glucose Dulbecco's modified Eagle's medium (Invitrogen, Waltham, MA) with 10% FBS and 1% penicillin‐streptomycin (Invitrogen, Waltham, MA, USA).

### Statistical Analyses

Statistical analysis was determined using GraphPad Prism 9 (GraphPad Software, Inc., La Jolla, CA, USA). Data are presented as mean values with error bars representing S.E.M. Two‐tailed unpaired Student's *t*‐test or Mann‐Whitney U test or one‐way ANOVA were used to determine differences between two or among three groups, respectively. Spearman correlation analysis was performed between the microbiota taxa and individual bile acids. Statistical outliers were determined using Grubb's test. Statistical difference was defined as *p* < 0.05.

## Conflict of Interest

The authors declare no conflict of interest.

## Author Contributions

J.T.X. and Y.L.H. contributed equally to this work. J.T.X. contributed to conceptualization, project administration, verification, writing–original draft. Y.L.H. validated the methodology and provided a writing review and editing. Y.H.Z., W.H.L., B.B.L., and L.X.Z. developed the methodology. X.C., Y.G., and X.M.Z. reviewed and edited the manuscript. H.Y.H. and X.W.L. provided conceptual support, and supervision, and contributed to reviewing and editing the manuscript.

## Supporting information



Supporting Information

## Data Availability

The data that support the findings of this study are available from the corresponding author upon reasonable request.
