# Supplementary material for: Clostridium Scindens Protects Against Vancomycin‐Induced Cholestasis and Liver Fibrosis by Activating Intestinal FXR‐FGF15/19 Signaling
Source: Adv Sci (Weinh). 2024 Dec 16;12(5):2406445. doi: 10.1002/advs.202406445 (PMC11791999; doi:10.1002/advs.202406445)
Supplement: Supplementary file 1 — Supporting Information [file ADVS-12-2406445-s001.docx]

***Clostridium Scindens* Protects Against Vancomycin Induced-cholestasis and Liver Fibrosis by Activating Intestinal FXR-FGF15/19 Signaling**

*Jintao Xiao^#^, Yanliang Hou^#^, Xingyang Luo, Yuhao Zhu, Wenhu Li, Bingbing Li, LinXiang Zhou, Xia Chen, Ying Guo, Xiaomei Zhang, Haiyue He^*^, Xiaowei Liu^*^*

J. Xiao, Y. Hou, X. Lou, Y. Zhu, W. Li, B. Li, L. Zhou, X. Zhang, H. He, X. Liu

Department of Gastroenterology, Xiangya Hospital, Central South University, Changsha 410008, Hunan, China

Email: haiyuehe@csu.edu.cn; liuxw@csu.edu.cn

X. Chen

Department of Clinical Laboratory, Xiangya Hospital, Central South University, Changsha 410008, Hunan, China.

Y. Guo,

Department of Clinical Pharmacology, Xiangya Hospital, Hunan Key Laboratory of Pharmacogenetics, Central South University, Changsha 410008, Hunan, China.

X. Liu

National Clinical Research Center for Geriatric Disorders, Xiangya Hospital, Central South University, Changsha, Hunan 410008, China.

**Supplemental material**

**Table S1** Demographic and clinical characteristics of control and CLD patients

| **Characteristics** | **Control (n=27)**  **Mean ± SEM** | **CLD (n=49)**  **Mean ± SEM** | **P value** |
| --- | --- | --- | --- |
| **Gender(Male%)** | 14/13 | 26/23 | ns |
| **Age** | 55.63±1.50 | 57.69±1.63 | ns |
| **TP** | 66.34 ±1.143 | 64.79 ±0.9360 | ns |
| **ALB** | 40.42 ± 0.6752 | 31.33 ± 0.72 | *p*＜0.0001 |
| **GLB** | 25.89 ± 0.69 | 33.39 ± 0.96 | *p*＜0.0001 |
| **TBIL** | 11.94 ± 0.89 | 21.62 ± 1.64 | *p*＜0.0001 |
| **DBIL** | 5.83 ± 0.47 | 11.22 ±0.93 | *p*＜0.001 |
| **IBIL** | 6.10 ± 0.45 | 9.91 ± 0.75 | *p*＜0.001 |
| **TBA** | 3.03 ± 0.55 | 33.64 ± 3.99 | *p*＜0.0001 |
| **ALT** | 17.89 ± 1.30 | 30.88 ± 2.67 | *p*＜0.001 |
| **AST** | 22.61 ± 1.14 | 42.31 ± 3.31 | *p*＜0.0001 |
| **ALP** | 70.20 ± 28.12 | 122.0 ± 7.65 | *p*＜0.001 |
| **GGT** | 21.41 ± 1.83 | 38.52 ± 3.00 | *p*＜0.001 |

**Table S2** The profiles of serum bile acids in control and CLD patients

| **Bile acids (ng/mL)** | **Control (n=27)**  **Mean ± SEM** | **CLD (n=49)**  **Mean ± SEM** | **P value** |
| --- | --- | --- | --- |
| **Total bile acids** | 6456.52 ± 1119.88 | 39385.23 ± 4732.40 | *p*＜0.0001 |
| **Primary bile acids** | 5133.33 ± 936.88 | 35817.25 ± 4326.84 | *p*＜0.0001 |
| **Secondary bile acids** | 3797.09 ± 2605.31 | 3601.31 ± 545.36 | *P*=0.93 |
| **Primary/secondary** | 14.42±4.444 | 17.88±3.353 | *p*＜0.05 |
| CA | 90.39 ± 26.99 | 423.57 ± 111.03 | *p*＜0.05 |
| GCA | 151.292 ± 47.47 | 3195.50 ± 471.61 | *p*＜0.0001 |
| TCA | 54.89 ± 8.72 | 4432.26 ± 743.97 | *p*＜0.0001 |
| CDCA | 542.07 ± 59.26 | 3080.00 ± 1025.39 | *p*=0.29 |
| GCDCA | 1622.62 ± 193.26 | 19927.60 ± 2863.63 | *p*＜0.0001 |
| TCDCA | 101.52 ± 15.12 | 5240.69 ± | *p*＜0.0001 |
| UDCA | 339.97 ± 65.88 | 324.25 ± 77.60 | *p* =0.27 |
| GUDCA | 248.97 ± 32.92 | 1359.10 ± 301.01 | *p*＜0.001 |
| TUDCA | 4.36 ± 0.99 | 129.80 ± 27.52 | *p*＜0.0001 |
| LCA | 18.60 ± 1.86 | 11.67 ± 1.47 | *p*＜0.01 |
| GLCA | 247.44 ± 36.69 | 3350.49 ± 485.77 | *p*＜0.0001 |
| TLCA | 0.17 ± 0.13 | 3.57 ± 0.83 | *p*＜0.05 |
| DCA | 190.42 ± 22.84 | 18.27 ± 6.19 | *p*＜0.0001 |
| GDCA | 192.58 ± 28.12 | 79.44 ± 20.76 | *p*＜0.0001 |
| TDCA | 23.91 ± 4.25 | 33.87 ± 13.22 | *p*＜0.01 |

**Table S3** List of antibodies used in this study

| **Antibody** | **Vendor** | **Catalog number** | **RRID** |
| --- | --- | --- | --- |
| β-Actin | Thermo Scientific | AM4302 | AB_437394 |
| α-SMA | Abcam | ab124964 | AB_11129103 |
| CYP7A1 | Santa Cruz Biotechnology | sc-518007 | AB_628835 |
| FGF15 | Santa Cruz Biotechnology | sc-514647 | AB_2827999 |
| HSP 90α/β | Santa Cruz Biotechnology | sc-7947 | AB_2121235 |

**Table S4** List of primer sequences used in this study

| **Primers** | **Forward** | **Reverse** |
| --- | --- | --- |
| *β-actin* | GGCTGTATTCCCCTCCATCG | CCAGTTGGTAACAATGCCATGT |
| *Acta2* | GTCCCAGACATCAGGGAGTAA | TCGGATACTTCAGCGTCAGGA |
| *Asbt* | GTCTGTCCCCCAAATGCAACT | CACCCCATAGAAAACATCACCA |
| *Bsep* | TCTGACTCAGTGATTCTTCGCA | CCCATAAACATCAGCCAGTTGT |
| *Baat* | GGAAACCTGTTAGTTCTCAGGC | GTGGACCCCCATATAGTCTCC |
| *Bacs* | ACCCTGGATCAGCTCCTGGAT | GTTCTCAGCTAGCAGCTTGG |
| *Col1a1* | GCTCCTCTTAGGGGCCACT | CCACGTCTCACCATTGGGG |
| *Col3a1* | CTGGTCAGCCTGGAGATAAG | ACCAGGACTACCACGTTCAC |
| *Cyp27a1* | CCTCACCTATGGGATCTTCATC | TTTAAGGCATCCGTGTAGAGC |
| *Cyp7a1* | GGGATTGCTGTGGTAGTGAGC | GGTATGGAATCAACCCGTTGTC |
| *Cyp7b1* | TAGCCCTCTTTCCTCCACTCATA | GAACCGATCGAACCTAAATTCCT |
| *Cyp8b1* | CCTCTGGACAAGGGTTTTGTG | GCACCGTGAAGACATCCCC |
| *E-* *cadherin* | CAGTTCCGAGGTCTACACCTT | TGAATCGGGAGTCTTCCGAAAA |
| *Fgf15* | ATGGCGAGAAAGTGGAACGG | CTGACACAGACTGGGATTGCT |
| *Fxr* | GCTTGATGTGCTACAAAAGCTG | CGTGGTGATGGTTGAATGTCC |
| *Ibabp* | GATCATCACAGAGGTCCAGC | CTCCATCTTCACGGTAGCCT |
| *Il-1β* | GAGAGCCGGGTGACAGTATC | TGACAAACTTCTGCCTGACG |
| *Il-6* | AGTTGCCTTCTTGGGACTGA | CAGAATTGCCATTGCACAAC |
| *Lxrα* | AGTGTAACAGGCGCTCCTTC | TTGGCAAAGTCTTCCCGGTT |
| *Lxrβ* | ACGATCTTTCTCCGACCAGC | CTGTCTCGTGGTTGTAGCGT |
| *Mmp2* | GCTGATACTGACACTGGTACTG | CAATCTTTTCTGGGAGCTC |
| *Muc2* | ATGCCCACCTCCTCAAAGAC | GTAGTTTCCGTGGGAACAGTGAA |
| *Ntcp* | ATCTGACCAGCATTGAGGCTC | CCGTCGTAGATTCCTTTGCTG |
| *Occludin* | CCTCCAATGGCAAAGTGAAT | CTCCCCACCTGTCGTGTAGT |
| *OSTα* | TGTTCCAGGTGCTTGTCATCC | CCACTGTTAGCCAAGATGGAGAA |
| *OSTβ* | GATGCGGCTCCTTGGAATTA | GGAGGAACATGCTTGTCATGAC |
| *Pxr* | GGGATAGGGTTACAGCACGA | TCTGAAAAACCCCTTGCATC |
| *Rorα* | AAATGAAACAATAACAACGAAGAC | TGGACTCTGCTGTTACCCG |
| *Shp* | TGGGTCCCAAGGAGTATGC | GCTCCAAGACTTCACACAGTG |
| *Tgf-β* | TTGCTTCAGCTCCACAGAGA | TGGTTGTAGAGGGCAAGGAC |
| *Tgr5* | CCTGGCAAGCCTCATCGTC | AGCAGCCCGGCTAGTAGTAG |
| *Timp1* | GCAACTCGGACCTGGTCATAA | CGGCCCGTGATGAGAAACT |
| *Tnf-α* | CGTCAGCCGATTTGCTATCT | CGGACTCCGCAAAGTCTAAG |
| *Vdr* | GAATGTGCCTCGGATCTGTGG | ATGCGGCAATCTCCATTGAAG |
| *16S* | ACTCCTACGGGAGGCAGCAGT | TATTACCGCGGCTGCTGGC |
| *C. scindens* | AACTTTCATGGCGGACACAC | AATATCGCAGAGTTCCGGGT |

**Table S5** List of the names of BAs and their corresponding abbreviations.

| **BAs** | **Abbreviation** |
| --- | --- |
| 3-dehydrocholic acid | 3_DHCA |
| 6-keto lithocholic acid | 6_ketoLCA |
| 7-dehydrocholic acid | 7_DHCA |
| 7-keto lithocholic acid | 7_ketoLCA |
| 12-dehydrocholic acid | 12_DHCA |
| 12-keto lithocholic acid | 12_KetoLCA |
| α-muricholic acid | α-MCA |
| β-cholic acid | β-CA |
| β-muricholic acid | β-MCA |
| ω-muricholic acid | ω-MCA |
| Allocholic acid | ACA |
| Cholic acid | CA |
| Chenodeoxycholic acid | CDCA |
| Deoxycholic acid | DCA |
| Deoxycholic acid-3- glucuronide | DCA-3-Glu |
| Dehydrocholic acid | DHCA |
| Glycocholic acid | GCA |
| Hyodeoxycholic acid | HDCA |
| Isoallolithocholic acid | IsoalloLCA |
| Lithocholic acid | LCA |
| Murocholic acid | MuroCA |
| Nordeoxycholic acid | NorDCA |
| Tauro-α-muricholic acid | T-α-MCA |
| Tauro-β-muricholic acid | T-β-MCA |
| Tauro-ω-muricholic acid | T-ω-MCA |
| Taurocholic acid | TCA |
| Taurochenodeoxycholic acid | TCDCA |
| Taurodeoxycholic acid | TDCA |
| Taurohyodeoxycholic acid | THDCA |
| Tauroursodeoxycholic acid | TUDCA |
| Ursocholic acid | UCA |
| Ursodeoxycholic acid | UDCA |

**
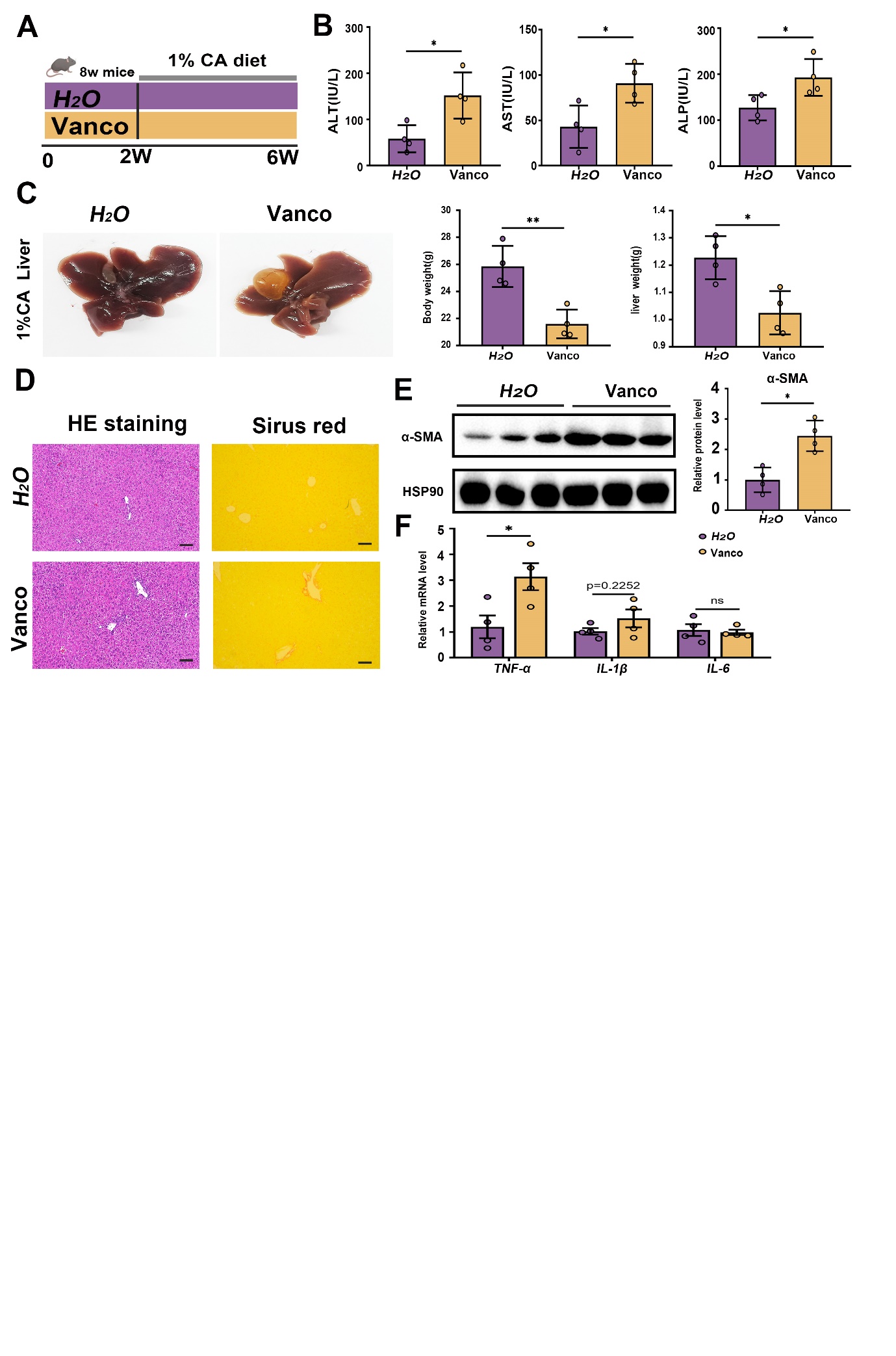
**

**Figure S1.** Vancomycin treatment aggravates fibrosis in 1% CA diet-fed mouse model

(A) Experimental scheme: C57BL/6J mice were pretreated with either vancomycin or regular water for 2 weeks, followed by a diet supplemented with 1% CA (w/w) diet for 4 weeks. Vanco-vancomycin. (B) Serum ALT, AST and ALP levels (C) Representative liver images of livers and total, ligated lobe weight and liver/body weight ratio. (D) Representative images of liver specimens stained with hematoxylin and eosin and Sirius red (scale bar, 100µm). (E) Representative immunoblots (left panel) and quantification of α-SMA (right panel) in the liver. (F) Relative mRNA expression of *Tnf-*α, *Il-1β* and *Il-6* in liver tissue. n=4 individuals/group. Data were expressed as mean ± SEM, ^*^*p*<0.05, ^**^*p*<0.01, ^***^*p*<0.001, ns-no significance.


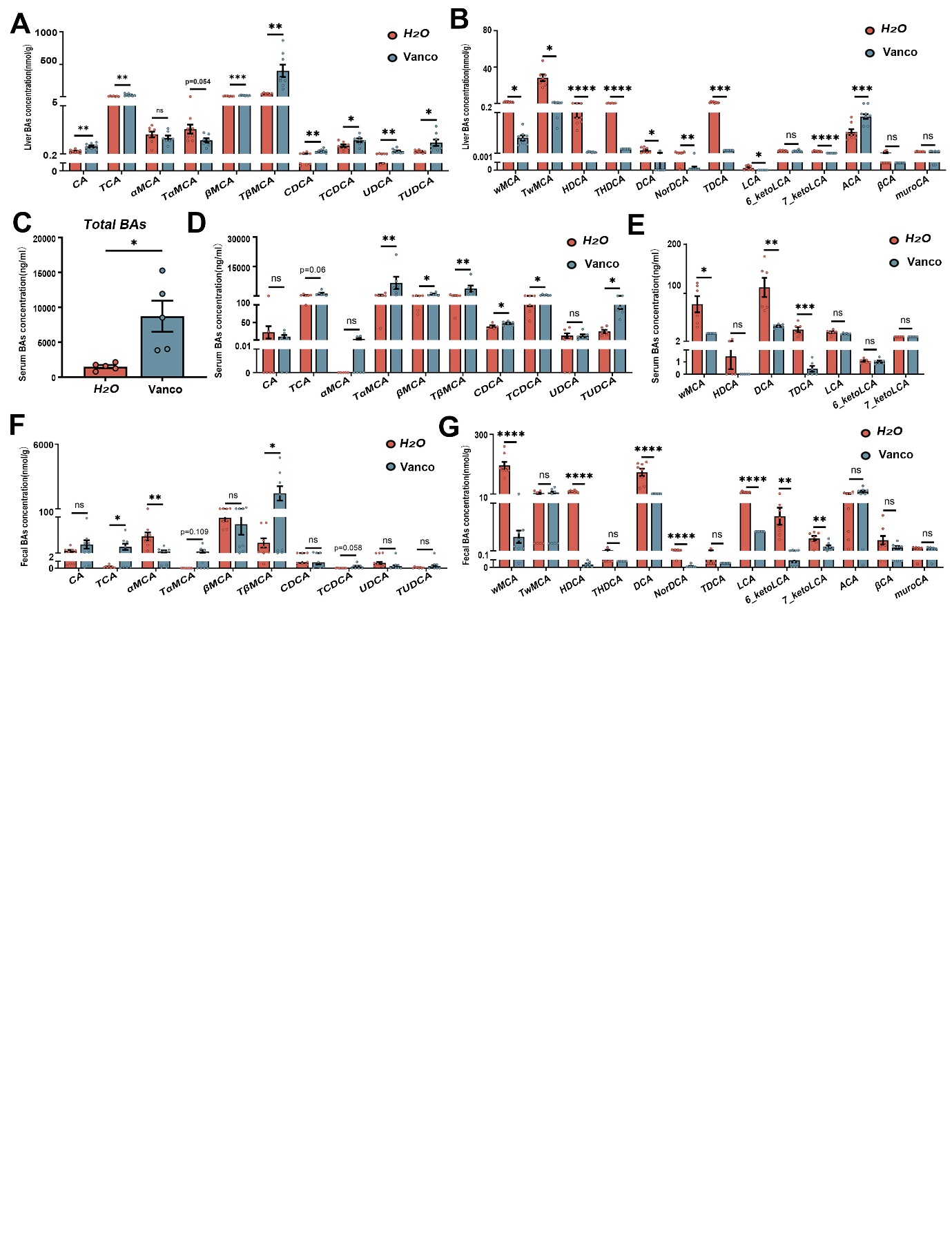


**Figure S2**. Vancomycin treatment promotes bile acid accumulation in the pBDL mouse model.

(A) Hepatic primary bile acid profile. (B) Hepatic secondary bile acid profile. (C) Concentration of total bile acid in serum. (D) Concentration of primary bile acid profile in serum. (E) Concentration of secondary bile acid profile in serum. (F) Primary bile acid profile in feces. (G) Secondary bile acid profile in feces. Data were expressed as mean ± SEM, n=5-6 individuals/group. ^*^*p*<0.05, ^**^*p*<0.01, ^***^*p*<0.001, ns-no significance.


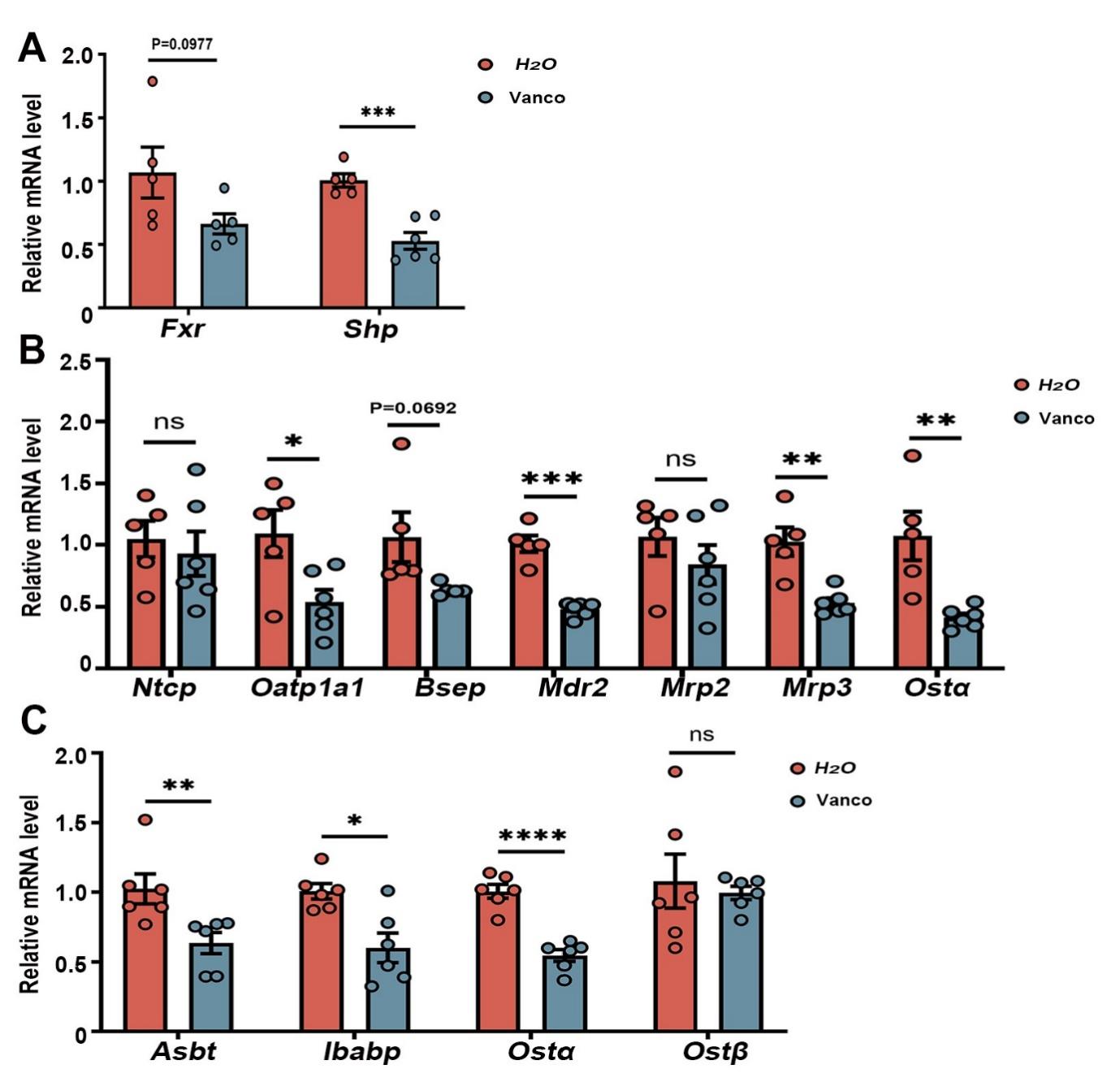


**Figure S3.** The influence of vancomycin treatment on hepatic FXR signaling, as well as hepatic and ileal bile acid transports in pBDL mouse model.

(A) Relative mRNA expression of *Fxr* and *shp* in liver tissue. (B) Relative mRNA expression of bile acid transports in liver tissue. (C) Relative mRNA expression of bile acid transports in the ileum. n=5-6 individuals/group. Data were expressed as mean ± SEM, ^*^*p*<0.05, ^**^*p*<0.01, ^***^*p*<0.001, ^****^*p*<0.0001, ns-no significance.

**
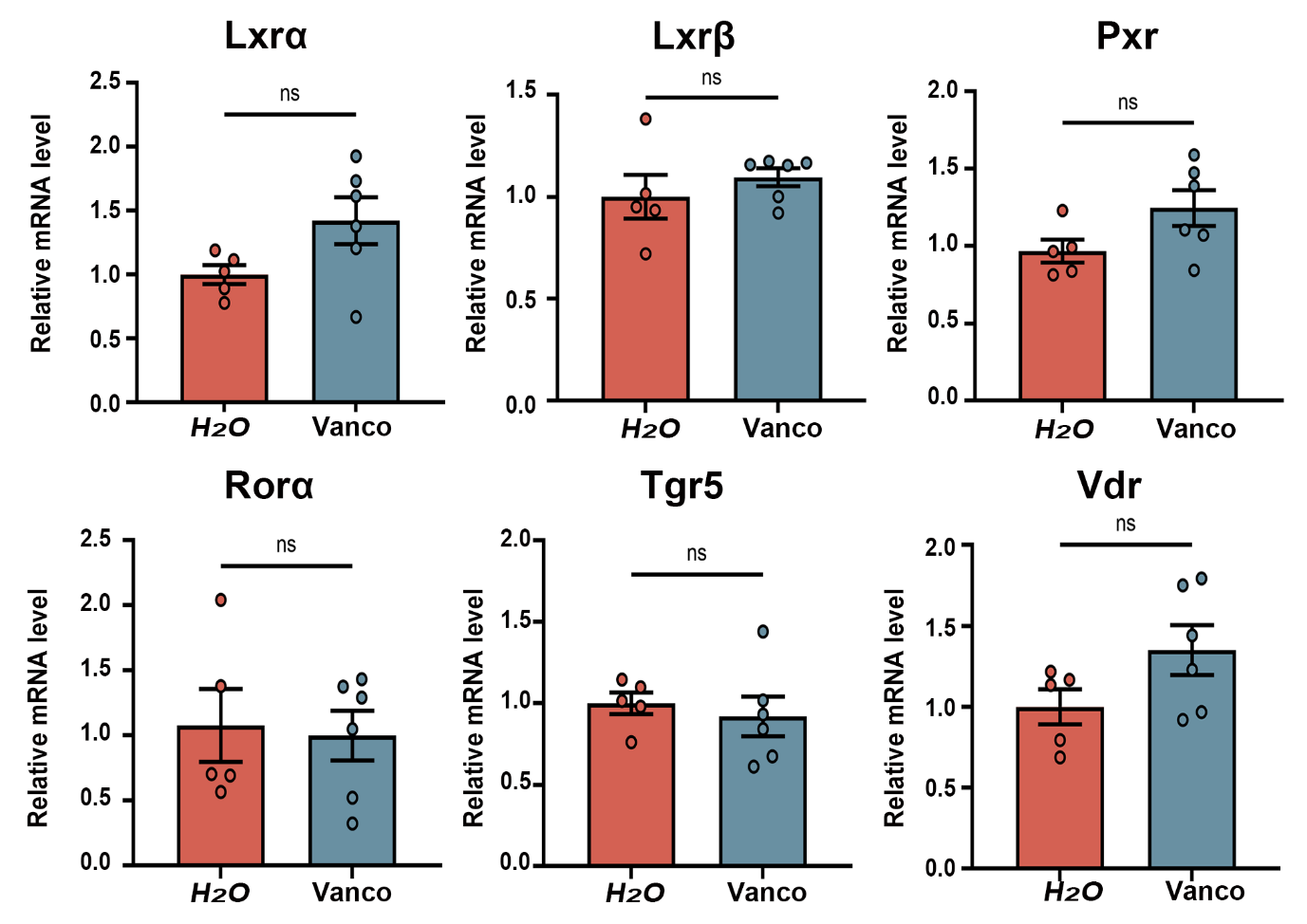
**

**Figure S4** The influence of vancomycin treatment on ileal bile acid receptor signaling in pBDL mouse model

Relative mRNA expression of bile aid-related receptors (*Lxrα, Lxrβ, Pxr, Rorα, Tgr5* and *Vdr*) in liver tissue. ns-no significance.

**
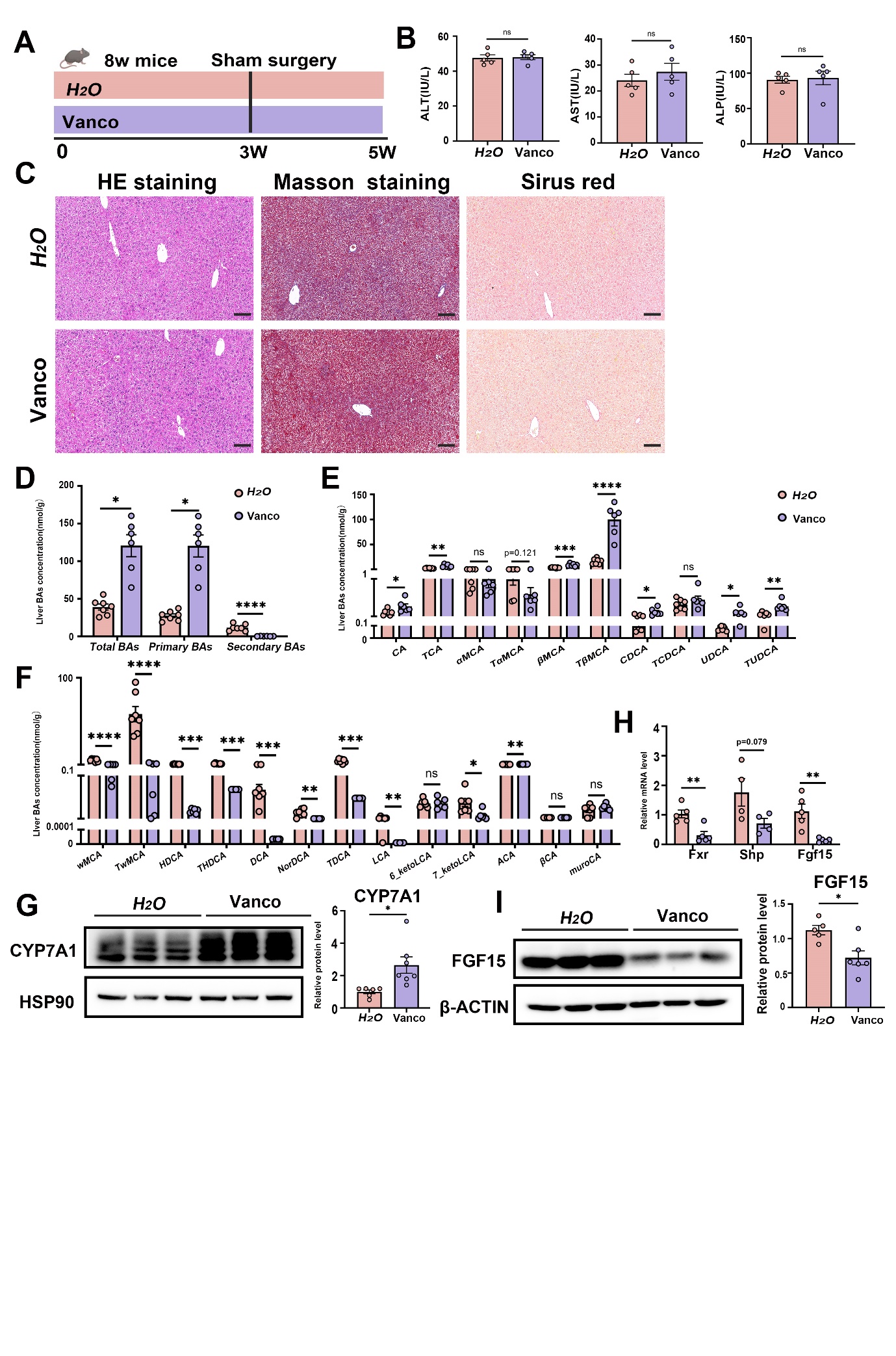
**

**Figure S5** Vancomycin treatment aggravates bile acid accumulation and suppresses intestinal FXR-FGF15/19 signaling in the absence of disease model

(A) Experimental scheme: C57BL/6J mice were pretreated with vancomycin or regular water for 3 weeks and followed by a sham operation for 2 weeks. (B) Serum ALT, AST and ALP levels. (C) Representative images of liver specimens stained with hematoxylin and eosin, Masson trichrome and Sirius red (scale bar, 100 µm). (D) Hepatic total, primary and secondary bile acid levels. (E) Hepatic primary bile acid profile. (F) Hepatic secondary bile acid profile. (G) Representative immunoblots (left panel) and quantification (right panel) of CYP7A1 in liver tissue. (H) Gene expression of *Fxr*, *Shp* and *Fgf15* in the ileum. (I) Representative immunoblots (left panel) and quantification (right panel) of FGF15 in ileum tissue. Data were expressed as mean ± SEM, n=5-6 individuals/group. ^*^*p*<0.05, ^**^*p*<0.01, ^***^*p*<0.001, ^****^*p*<0.0001; ns-no significance.


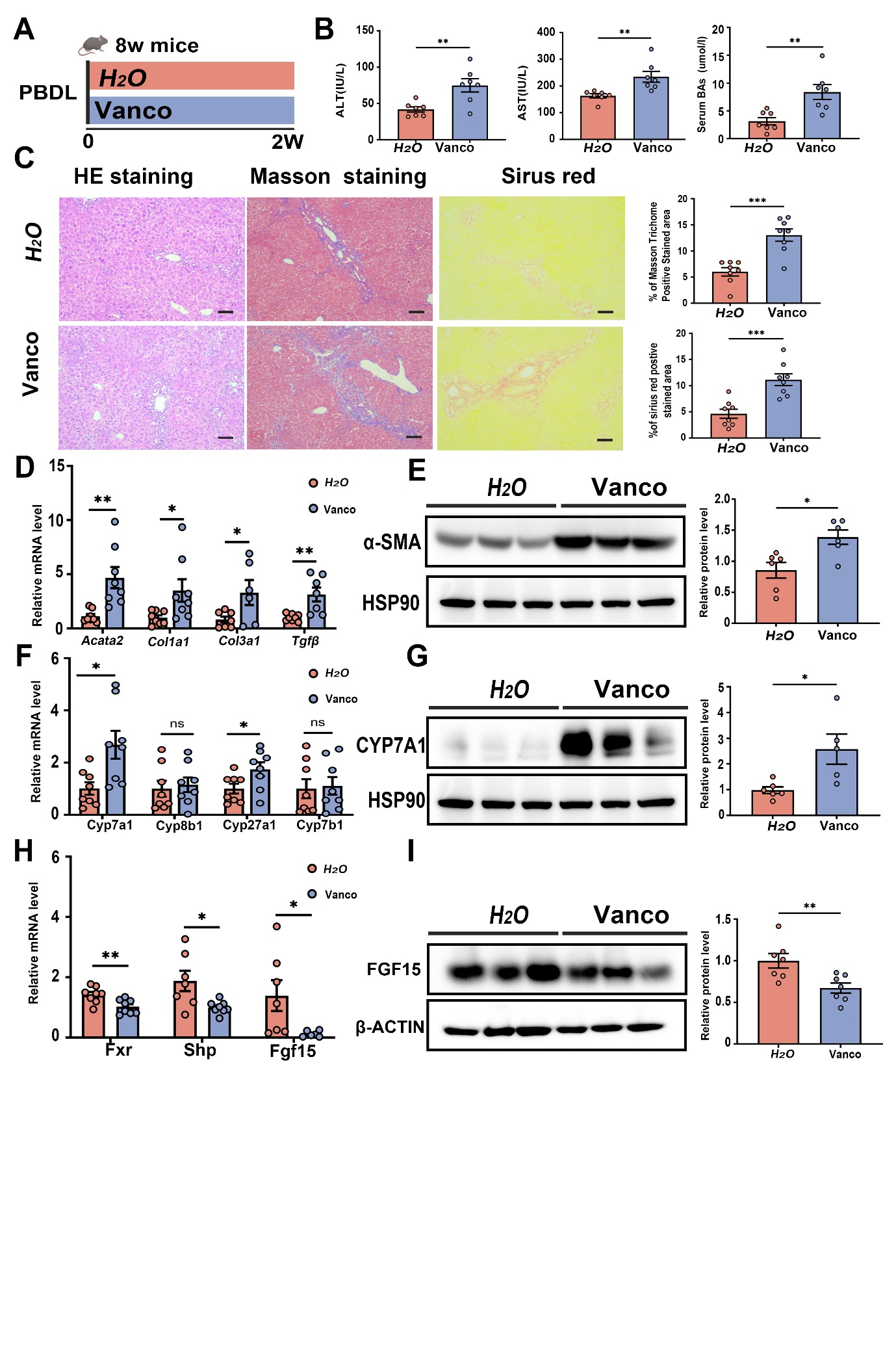


**Figure S6** Vancomycin treatment following pBDL exacerbates bile acid accumulation and liver fibrosis by inhibiting intestinal FXR-FGF15 signaling

(A) Experimental scheme: C57BL/6J mice were subjected to pBDL operation and followed by vancomycin administration for 2 weeks. (B) Serum ALT, AST and bile acid levels. (C) Representative images of liver specimens stained with hematoxylin and eosin, Masson trichrome and Sirius red (scale bar, 100 µm). The bar graph represents the average percentage of Masson trichrome or Sirius red positive area per field. (D) Hepatic mRNA expression of liver fibrosis-related genes. (E) Representative immunoblots (left panel) and quantification (right panel) of α-SMA in liver tissue. Hepatic total, primary and secondary bile acid levels. (F) Hepatic mRNA expression of bile acid synthetic genes. (G) Representative immunoblots (left panel) and quantification (right panel) of CYP7A1 in liver tissue. (H) Gene expression of *Fxr*, *Shp* and *Fgf15* in the ileum. (I) Representative immunoblots (left panel) and quantification (right panel) of FGF15 in ileum tissue. Data were expressed as mean±SEM, n=6-8 individuals/group. ^*^*p*<0.05, ^**^*p*<0.01, ^***^*p*<0.001; ns-no significance.

**
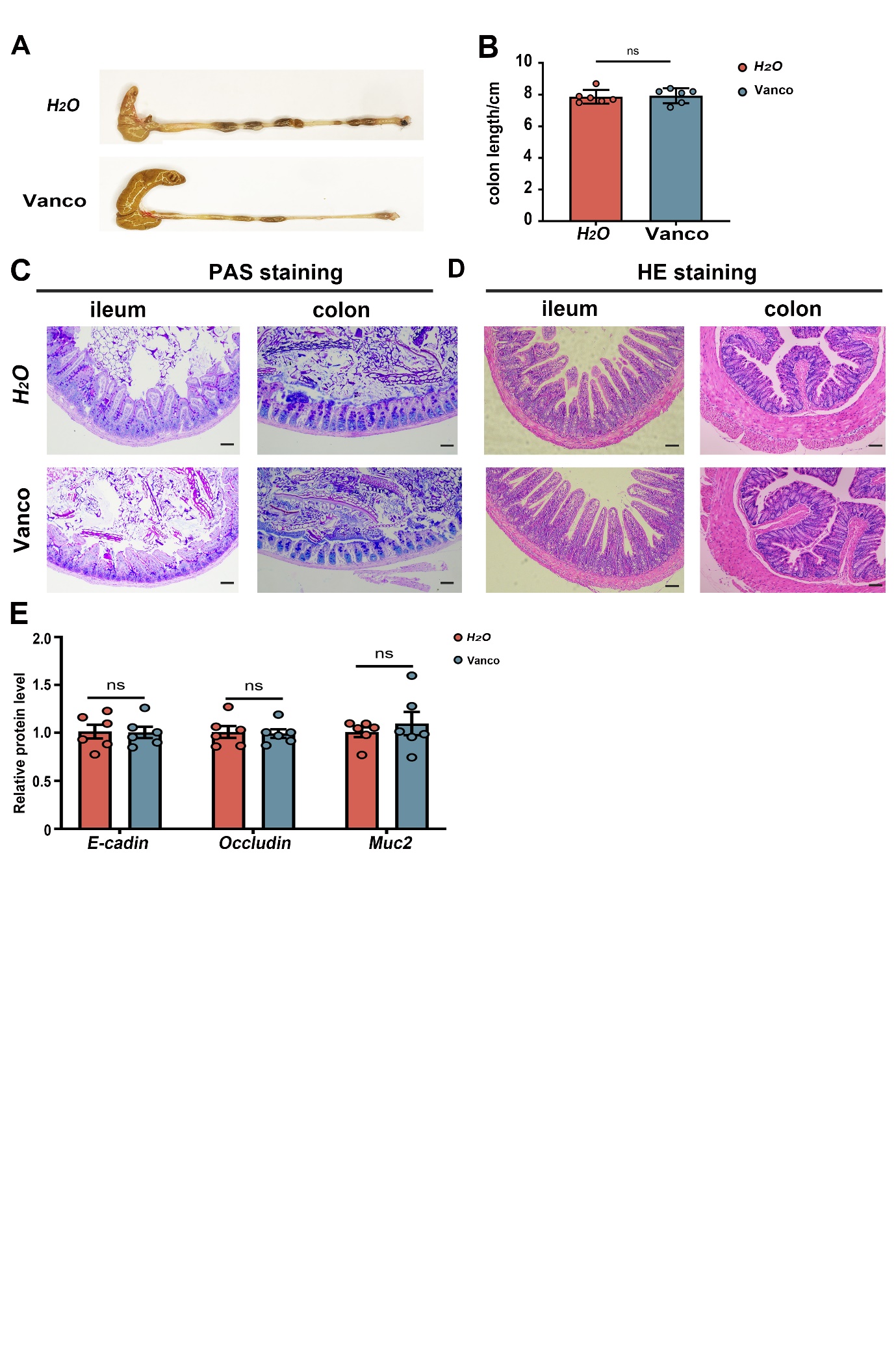
**

**Figure S7.** Influence of vancomycin treatment on intestinal barrier function

(A) Representative images of resected colons of mice. (B) The length of the colon of mice. Representative images of the colon tissue stained with periodic acid-Schiff (PAS) (C) and hematoxylin and eosin (H&E) staining (D), scale bar, 200µm. (E) Relative mRNA expression of *E-cadherin*, *Occludin* and *Muc2* in the ileum. n=5-6 individuals/group. Data were expressed as mean ± SEM, ns-no significance.

**
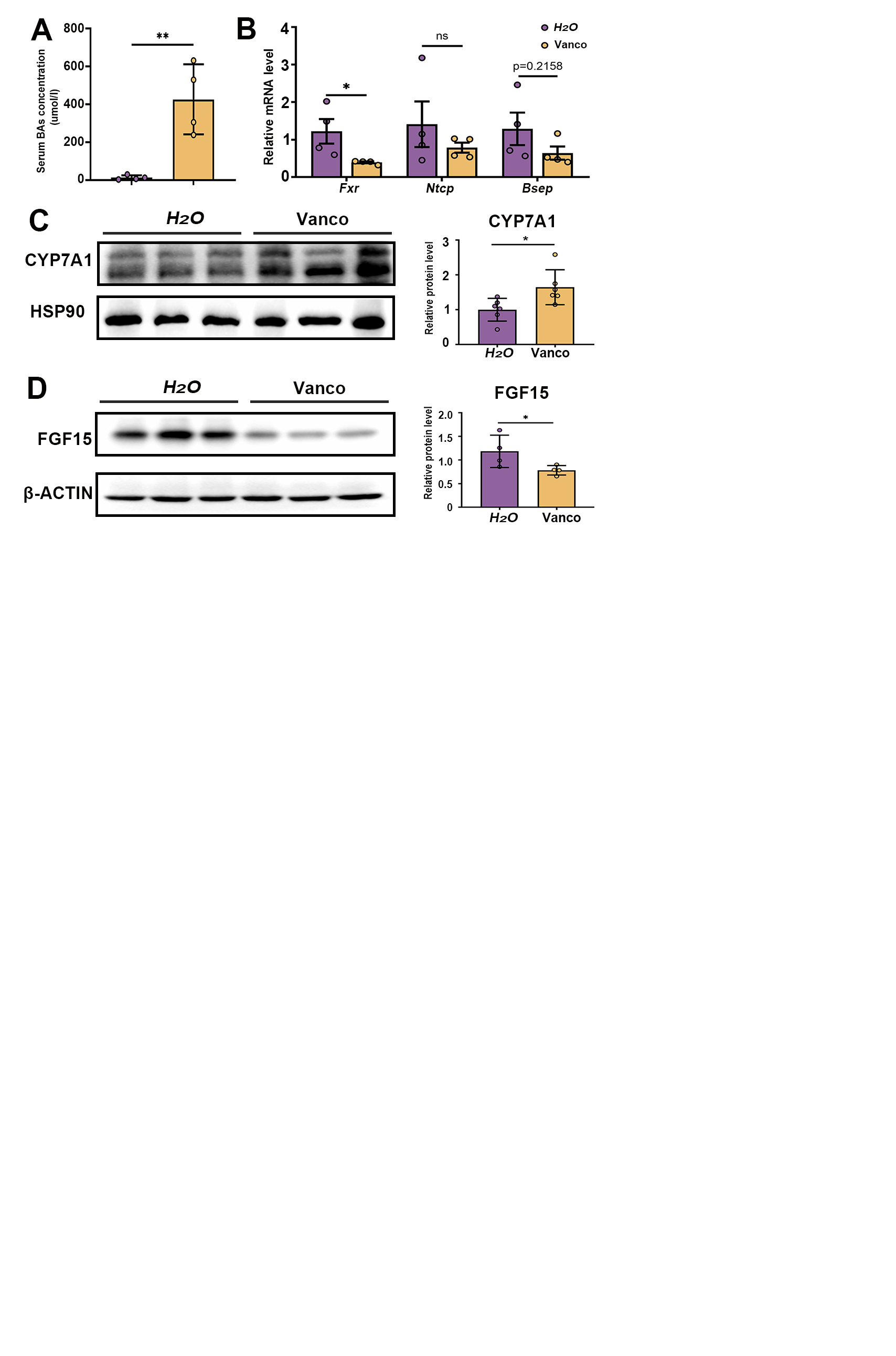
Figure S8.** Vancomycin treatment inhibits FXR-FGF15/19 signaling in 1% CA diet-fed mouse model

(A) Concentrations of total bile acids in serum. (B) Relative mRNA expression of *Fxr*, *Ntcp* and *Bsep* in liver tissue. (C) Representative immunoblots (left panel) and quantification (right panel) of CYP7A1 in the liver. (D) Representative immunoblots (left panel) and quantification (right panel) of FGF15 in the ileum. n=4-6 individuals/group. Data were expressed as mean ± SEM, ^*^*p*<0.05, ^**^*p*<0.01, ^***^*p*<0.001, ns-no significance.

**
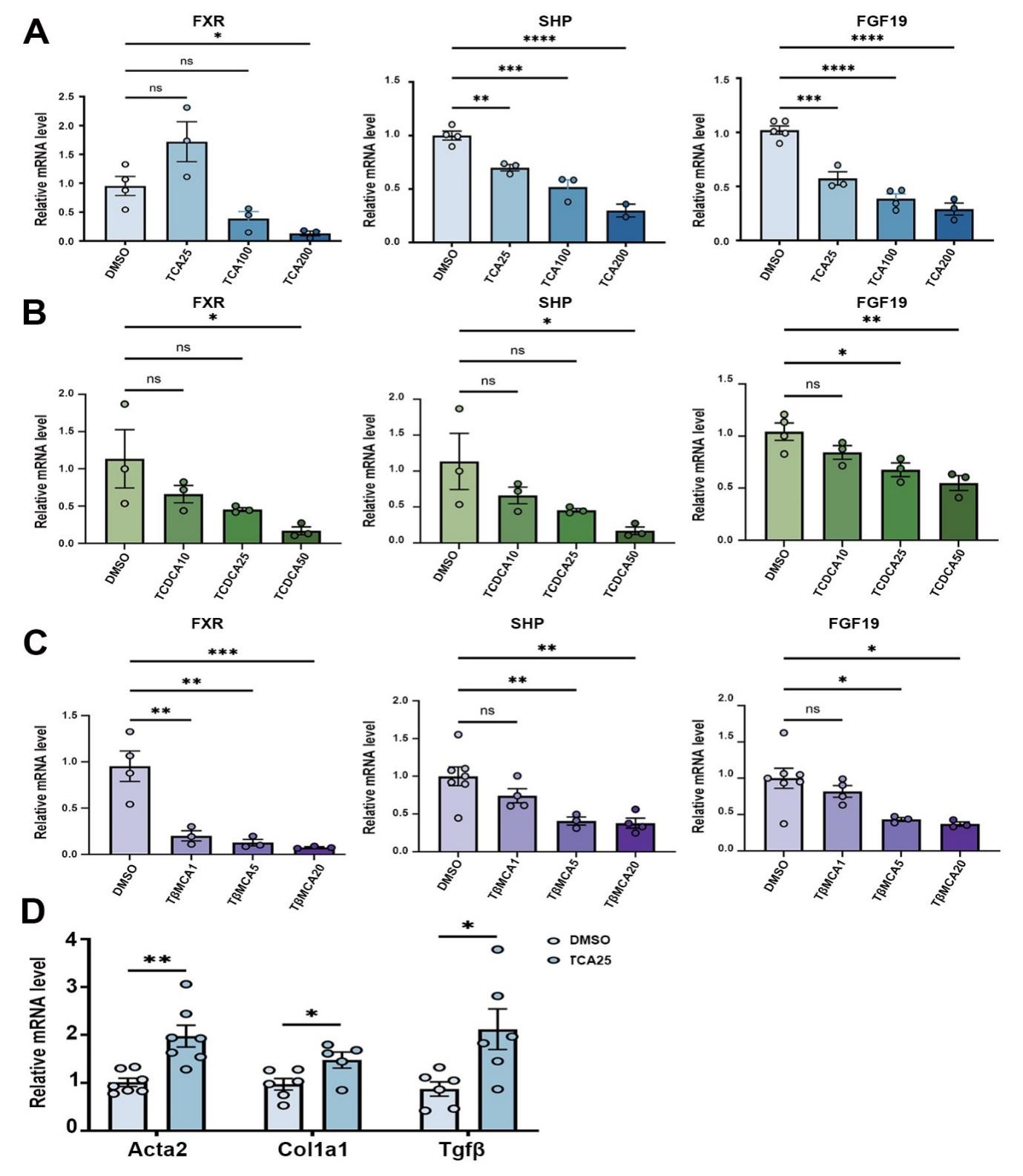
**

**Figure S9.** Taurine-conjugated primary bile acids inhibit intestinal FXR-FGF15/19 signaling and promote HSC activation in vitro

Relative mRNA expression of *Fxr, Shp* and *Fgf19* in Caco2 cells treated with varying concentrations of TCA (A), TCDCA (B) and T-β-MCA (C) (n=3-6 biologically independent cells). (D) Relative mRNA expression of *Acta2*, *Col1a1* and *Tgf-β* in LX2 cells treated with TCA. Data were expressed as mean ± SEM, ^*^*p*<0.05, ^**^*p*<0.01, ^***^*p*<0.001, ^****^*p*<0.001. ns-no significance.

**
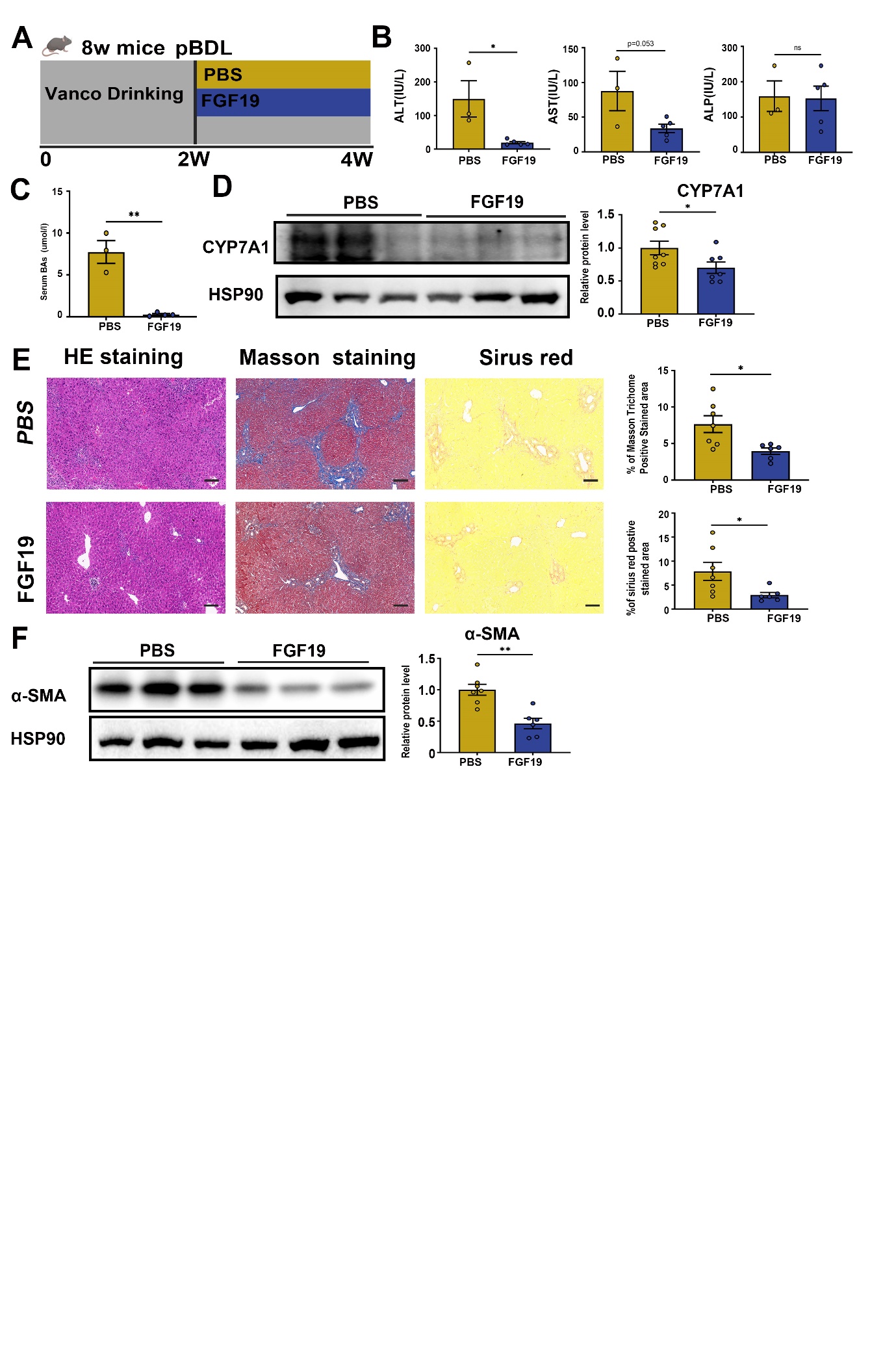
**

**Figure S10.** Recombinant FGF-19 protein reverses vancomycin-induced liver fibrosis

(A) Experimental scheme: After 2 weeks of vancomycin treatment, all mice were subjected to pBDL and then intraperitoneal injected with recombinant FGF-19 protein or vehicle (PBS). (B) The levels of ALT, AST and ALP in serum. (C) Concentrations of total bile acids in serum. (D) Representative immunoblots (left panel) and quantification (right panel) of CYP7A1 in the liver. (E)Representative images of liver specimens stained with hematoxylin and eosin, Masson’s trichrome and Sirius red (scale bar, 100µm). The bar graph represents the average percentage of Masson trichrome or Sirius red positive area per field. (F) Representative immunoblots (left panel) and quantification (right panel) of α-SMA in the liver. n=6-7 individuals/group. Data were presented as mean ± SEM, ^*^*p*<0.05, ^**^*p*<0.01; ns-no significance.
